# Decomposable and Essentially Univariate Mass-Action Systems: Extensions of the Deficiency One Theorem

**DOI:** 10.1007/s00332-026-10278-4

**Published:** 2026-06-02

**Authors:** Abhishek Deshpande, Stefan Müller

**Affiliations:** 1 Center for Computational Natural Sciences and Bioinformatics, International Institute of Information Technology Hyderabad, Hyderabad, Telangana 500032 India; 2 Faculty of Mathematics, University of Vienna, Oskar-Morgenstern-Platz 1, 1090 Wien, Austria

**Keywords:** Reaction networks, Mass-action kinetics, Existence of a unique positive equilibrium, Parametrized systems of polynomial equations, Monomial dependency, Dependency one theorem, 12D10, 26C10, 92C42

## Abstract

The classical and extended deficiency one theorems by Feinberg apply to reaction networks with mass-action kinetics that have independent linkage classes or subnetworks, each with a deficiency of at most one and exactly one absorbing strong component. The theorems assume the existence of a positive equilibrium and guarantee the existence of a unique positive equilibrium in every stoichiometric compatibility class. In our work, we use the *monomial dependency* which extends the concept of deficiency. First, we provide a dependency one theorem for parametrized systems of polynomial equations that are essentially univariate and decomposable. As our main result, we present a corresponding theorem for mass-action systems, which permits subnetworks with arbitrary deficiency and arbitrary number of absorbing strong components. Finally, to complete the picture, we derive the extended deficiency one theorem as a special case of our more general dependency one theorem.

## Introduction

Many systems in chemistry and biology (particularly in ecology and epidemiology) and also in economics and engineering are modeled as polynomial or power-law dynamical systems. These models can be formulated as *reaction networks* with mass-action or generalized *mass-action kinetics*. They capture complex dynamic behaviors, such as multistationarity, oscillations, and chaos, and the corresponding bifurcations.

The most prominent results of (chemical) reaction network theory, as founded in the 1970’s by Horn, Jackson, and Feinberg, are the *deficiency zero* and *one theorems* (for mass-action systems) (Horn and Jackson 1972; Horn 1972; Feinberg 1972, 1987, 1995b). In essence, the concept of deficiency captures affine dependencies between *complexes* (representing the left- and right-hand sides of the reactions). The deficiency zero theorem assumes *weak reversibility* (and deficiency zero) and guarantees the existence of a unique positive equilibrium in every *stoichiometric compatibility class* (invariant subspace) and for all *rate constants* (system parameters); moreover, it ensures the asymptotic stability of this equilibrium. The deficiency one theorem has several assumptions. It applies to mass-action systems with *independent* linkage classes or subnetworks, *each* with a deficiency of at most one and exactly one absorbing strong component. Additionally, the theorem assumes the existence of a positive equilibrium (for given rate constants) and guarantees the existence of a unique positive equilibrium in every compatibility class (but does not address stability). Finally, weak reversibility ensures the existence of a positive equilibrium.

Both, the deficiency zero and one theorems, have been difficult to improve upon. Only after 2010, the deficiency zero theorem has been extended, namely from mass-action to *generalized* mass-action kinetics (Müller and Regensburger 2012, 2014; Müller et al. 2016, 2019; Craciun et al. 2019; Boros et al. 2020; Müller and Regensburger 2024). In this work, we will extend the deficiency one theorem, even in the setting of mass-action kinetics.

The deficiency one theorem was first fully stated in 1987 (Feinberg 1987), but was not proved until 1995 (Feinberg 1995a). Its extension from independent linkage classes to independent subnetworks was mentioned in (Feinberg 1987), and a proof was outlined in (Feinberg 1995a). Since then, the upper bound of one for both the deficiency and the number of absorbing strong components (per independent linkage class or subnetwork) has not been addressed. Only the assumption of existence (of a positive equilibrium) has been investigated further (Boros 2010, 2012, 2013a, b). In (Boros 2010, 2012), Boros provides an equivalent condition for the existence of a positive equilibrium for reaction networks that satisfy the assumptions of the deficiency one theorem. In (Boros 2013a), he characterizes single linkage-class, deficiency one mass-action systems for which a positive equilibrium exists for all or some rate constants. Finally, in (Boros 2013b), Boros proves the existence of a positive equilibrium within every stoichiometric compatibility class for weakly reversible, deficiency one mass-action systems. Using different methods, this result can be further extended to weakly reversible (not necessarily deficiency one) mass-action systems (Boros 2019).

In this work, we extend the validity of the deficiency one theorem to a much broader class of mass-action systems. First, we consider independent subnetworks (instead of independent linkage classes) from the outset. Second, we do not assume that the subnetworks have a deficiency of at most one. Instead, we use the *monomial dependency* which generalizes the concept of deficiency. Specifically, dependency accounts for the fact that non-source vertices in a reaction network do not contribute monomials to the polynomial equations for the equilibria. As a consequence, the dependency is often smaller than the deficiency. Third, we do not assume that the subnetworks have exactly one absorbing strong component.

We illustrate our results in a series of examples in Sect. 8. Here, we consider Example 2, given by the reaction network

$$ 0 \leftarrow X_1 \rightleftarrows X_1+X_2 \rightarrow X_2 \rightleftarrows 3\, X_1 $$

or, equivalently, by the “embedded” graph 
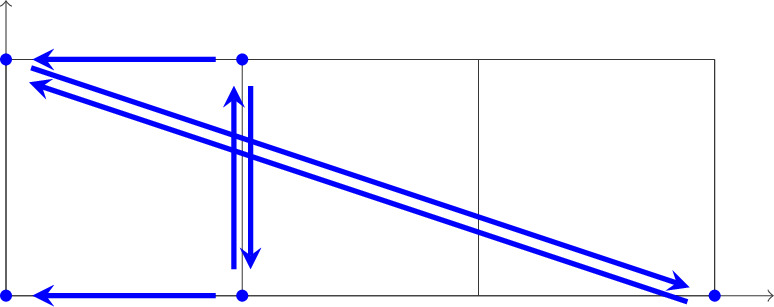
 and assume mass-action kinetics. The network has one independent linkage class/subnetwork. Hence, its deficiency is $\delta = |V| - 1 - \dim (S)=5-1-2 = 2$, where *V* is the set of vertices and *S* is the stoichiometric subspace (the linear span of the differences of complexes). However, its dependency is $d = |V_s| - 1 - \dim (L) = 4-1-2 = 1$, where $V_s$ is the set of source vertices and *L* is the monomial difference subspace (the linear span of the differences of source complexes). Moreover, this network has two absorbing strong components, one singleton (complex 0) and one non-singleton (with complexes $X_2$ and $3\, X_1$). Clearly, it does not satisfy the conditions of the deficiency one theorem. Still, our dependency one results for mass-action systems, Theorem 14 and Corollary 15, can be applied.

Technically, we treat positive equilibria of mass-action systems as *parametrized* systems of *polynomial equations* with *classes* and apply recent fundamental results for such systems (Müller and Regensburger 2026). Specifically, their solution set is essentially the solution set on the *coefficient polytope* (modulo an exponential fiber involving the *monomial difference subspace* *L*). We assume (i) the reaction network can be decomposed into independent subnetworks, (ii) the equation system is *decomposable* in the terminology of (Müller and Regensburger 2026), (iii) the subsystems (classes) have *monomial dependency* one and hence are univariate, and (iv) $L=S$ (or $L=K$, where *K* is the kinetic subspace). Then, there exists a unique positive equilibrium in every stoichiometric (or kinetic) compatibility class. Note that, even if $K=S$, the subnetworks need not have exactly one absorbing strong component.

We first provide a *dependency one theorem* for one class, Theorem 5, using the fundamental result from (Müller and Regensburger 2026), Theorem 2. Next, we extend it to *decomposable systems* (with several classes) and obtain Theorem 8. By applying the latter to reaction networks (and using Birch’s theorem), we arrive at a dependency one theorem for mass-action systems, Theorem 14. Finally, this allows us to provide a modular proof of the extended deficiency one theorem, Theorem 18, and hence of the classical deficiency one theorem. 
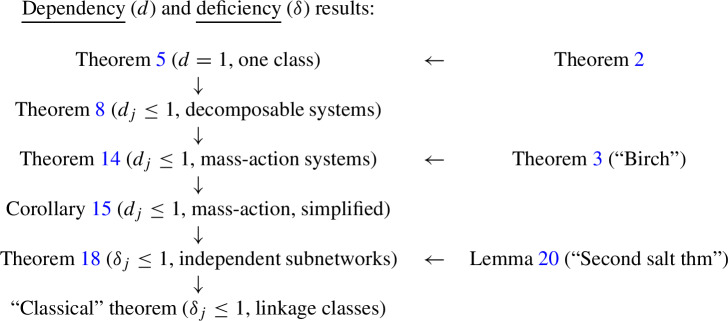


### Organization of the Work

In Sect. 2, we recall the relevant geometric objects corresponding to parametrized systems of generalized polynomial equations (with classes) including the coefficient polytope and the monomial dependency and difference subspaces. In Sect. 3, we provide sufficient conditions for the unique existence of a solution on the coefficient polytope, along with an equivalent condition for its existence. In Sect. 4, we introduce basic notions for reaction networks with mass-action kinetics, and in Sect. 5, we decompose a reaction network into subnetworks (as a pre-processing step) and finally into independent subnetworks. In Sect. 6, we present our main result, the dependency one theorem for mass-action systems, and in Sect. 7, we derive the extended deficiency one theorem as a special case. Finally, in Sect. 8, we provide three examples where the conditions of the deficiency one theorem are not satisfied, but the dependency one theorem can be applied (to conclude the unique existence of a positive equilibrium within every stoichiometric compatibility class for some or all rate constants).

#### Notation

For vectors $x, y \in \mathbb {R}^n$, we denote their scalar product by $x \cdot y \in \mathbb {R}$ and their component-wise (Hadamard) product by $x \circ y \in \mathbb {R}^n$. We denote the vector with all entries equal to one by $1_n \in \mathbb {R}^n$ and the identity matrix by ${{\,\textrm{Id}\,}}_n \in \mathbb {R}^{n \times n}$.

We denote the positive (non-negative) real numbers by $\mathbb {R}_>$ $(\mathbb {R}_\ge )$. For $x \in \mathbb {R}^n_>$ and $y \in \mathbb {R}^n$, we define the monomial $x^y = \prod _{i=1}^n (x_i)^{y_i} \in \mathbb {R}_>$; and for $Y = (y^1,\ldots ,y^m) \in \mathbb {R}^{n \times m}$, we define the vector of monomials $x^Y \in \mathbb {R}^m_>$ via $(x^Y)_j = x^{y^j}$.

For $x \in \mathbb {R}^n$, we define ${{\,\textrm{e}\,}}^x = (e^{x_1},e^{x_2}, \ldots , e^{x_n})^\textsf{T}\in \mathbb {R}^n_>$; and for $x \in \mathbb {R}^n_>$, we define $\ln (x) = \left( \ln (x_1),\ln (x_2), \ldots , \ln (x_n) \right) ^\textsf{T}\in \mathbb {R}^n$.

## Previous Results on Polynomial Systems

In order to state Theorem 2 below for a parametrized system of generalized polynomial equations, we introduce geometric objects and auxiliary matrices as defined in (Müller and Regensburger 2023, 2026).

### Definition 1

For the positive variable vector $x \in \mathbb {R}_>^n$, a *coefficient matrix* $A \in \mathbb {R}^{n' \times m}$, an *exponent matrix* $B \in \mathbb {R}^{n \times m}$, and a positive *parameter vector* $c \in \mathbb {R}_>^m$, we define the parametrized system of generalized polynomial equations

$$\begin{aligned} A \, (c \circ x^B)=0. \end{aligned}$$

(i) We call $C = \ker A \cap \mathbb {R}_>^m$ the *coefficient cone*. Its closure $\overline{C} = \ker A \cap \mathbb {R}_\ge ^m$ is a polyhedral cone, called an s-cone (subspace cone) in (Müller and Regensburger 2016). As a necessary condition for the existence of solutions, *C* must be non-empty.
(ii) We assume that $$ A = \begin{pmatrix} A_1&\ldots&A_\ell \end{pmatrix} \in \mathbb {R}^{n' \times m} $$ with $\ell \ge 1$ blocks $A_j \in \mathbb {R}^{n' \times m_j}$ (and hence $m_1+\ldots +m_\ell = m$) such that the kernel of *A* is the direct product of the kernels of $A_j$, that is, $\ker A = \ker A_1 \times \cdots \times \ker A_\ell .$ Accordingly, $$ B = \begin{pmatrix} B_1&\ldots&B_\ell \end{pmatrix} \in \mathbb {R}^{n \times m} $$ with $\ell$ blocks $B_j \in \mathbb {R}^{n \times m_j}$ and $$ c^\textsf{T}= \begin{pmatrix} (c^1)^\textsf{T}&\ldots&(c^\ell )^\textsf{T}\end{pmatrix} \in \mathbb {R}^m_> $$ with $c^j\in \mathbb {R}^{m_j}_>$. The decomposition of $\ker A$ induces a partition of the indices $\{1,\ldots ,m\}$ into $\ell$ *classes*. In particular, the columns of $B=(b^1,\ldots ,b^m)$ and hence the monomials $x^{b^j}$, $j=1,\ldots ,m$ are partitioned into classes.
(iii) We introduce the direct product $\Delta = \Delta ^{m_1-1} \times \cdots \times \Delta ^{m_\ell -1}$ of the standard simplices $\Delta ^{m_j-1} = \{ y \in \mathbb {R}^{m_j}_\ge \mid 1_{m_j} \cdot y = 1 \}$ and define the bounded set $P = C \cap \Delta .$ Clearly, $P = P_1 \times \cdots \times P_\ell$ with $P_j = C_j \cap \Delta ^{m_j-1}.$ We call *P* the *coefficient polytope*. In fact, *P* is a polytope without boundary. Strictly speaking, only its closure ${\overline{P}}$ is a polytope.
(iv) Let $$I_m = \begin{pmatrix} {{\,\textrm{Id}\,}}_{m-1} \\ -1_{m-1}^\textsf{T}\end{pmatrix} \in \mathbb {R}^{m \times (m-1)}$$, which can be seen as the incidence matrix of a star-shaped graph with vertices $\{1,\ldots ,m\}$ and root *m*. We introduce the $\ell \times \ell$ block-diagonal (incidence) matrix
  $$\begin{aligned} I = \begin{pmatrix} I_{m_1} & & 0 \\  & \ddots & \\ 0 & & I_{m_\ell } \end{pmatrix} \in \mathbb {R}^{m \times (m-\ell )} \end{aligned}$$
  with blocks $I_{m_j} \in \mathbb {R}^{m_j \times (m_j-1)}$ and the “monomial difference” matrix $M = B \, I \in \mathbb {R}^{n\times (m-\ell )}.$ Clearly, $$ M = \begin{pmatrix} B_1 I_{m_1}&\ldots&B_\ell I_{m_\ell } \end{pmatrix} $$ is generated by taking the differences between the first $m_j-1$ columns of $B_j$ and its last column, for $j=1,\ldots ,\ell$, and hence $L = {{\,\textrm{im}\,}}M \subseteq \mathbb {R}^n$ is the sum of the linear subspaces associated with the affine spans of the columns of *B* in the $\ell$ classes. We call *L* the *monomial difference subspace*. Further, we call $d = \dim (\ker M)$ the *monomial dependency*. It can be determined as $d = m-\ell -\dim L,$ cf. (Müller and Regensburger 2026, Proposition 1).
(v) We introduce the $\ell \times \ell$ block-diagonal “Cayley” matrix
  $$\begin{aligned} J = \begin{pmatrix} 1_{m_1}^\textsf{T}& & 0 \\  & \ddots & \\ 0 & & 1_{m_\ell }^\textsf{T}\end{pmatrix} \in \mathbb {R}^{\ell \times m} \end{aligned}$$
  with blocks $1_{m_j}^\textsf{T}\in \mathbb {R}^{1 \times m_j}$ and the matrix $$ \mathcal {B} = \begin{pmatrix} B \\ J \end{pmatrix} \in \mathbb {R}^{(n+\ell ) \times m}. $$ We call $D = \ker \mathcal {B} \subset \mathbb {R}^m$ the *monomial dependency subspace*. It records affine dependencies between the columns of *B* within the $\ell$ classes. In fact, $\dim D = d$, cf. (Müller and Regensburger 2026, Lemma 4).
(vi) Finally, we introduce the “exponentiation” matrix $E = I M^* \in \mathbb {R}^{m \times n}$, where $M^* \in \mathbb {R}^{(m-\ell ) \times n}$ is a generalized inverse of *M*.

We can now state the main result of our previous work on parametrized systems of polynomial inequalities (Müller and Regensburger 2026), instantiated for equations, cf. (Müller and Regensburger 2023).

### Theorem 2

(Müller and Regensburger 2023, Theorem 1) Consider the parametrized system of generalized polynomial equations $A \, (c \circ x^B) = 0$. The solution set $Z_c = \{ x \in \mathbb {R}^n_> \mid A \, (c \circ x^B) = 0 \}$ can be written as

$$ Z_c = \{ (y\, \circ \, c^{-1})^E \mid y \in Y_c \} \circ {{\,\textrm{e}\,}}^{L^\perp }, $$

where

$$ Y_c = \{y \in P \mid y^z = c^z \text { for all } z\in D\} $$

is the solution set on the coefficient polytope *P*.

Theorem 2 can be read as follows: In order to determine the solution set $Z_c$, first determine the solution set on the coefficient polytope, $Y_c$. Recall that the coefficient polytope *P* is determined by the coefficient matrix *A*, and the dependency subspace *D* is determined by the exponent matrix *B* (and the classes). To a solution $y \in Y_c$, there corresponds the actual solution $x = (y \circ c^{-1})^E \in Z_c$. In fact, if (and only if) $\dim L < n$, then $y \in Y_c$ corresponds to an exponential manifold of solutions, $x \circ {{\,\textrm{e}\,}}^{L^\perp } \subseteq Z_c$. Strictly speaking, existence of a unique solution corresponds to $|Y_c|=1$ and $\dim L = n$ (that is, $L^\perp = \{0\}$).

### Theorem 3

(“Birch’s theorem”) Let $x_0,x^*\in \mathbb {R}^n_>$ and let $S\subseteq \mathbb {R}^n$ be a subspace. Then we have

$$ |(x_0 + S) \cap (x^*\circ S^{\perp })|=1. $$

Theorem 3 was originally proved by Birch (Birch, 1963) and reproved by Horn and Jackson (Horn and Jackson 1972, Lemma 4B) in the context of reaction networks with mass-action kinetics. Motivated by applications, Birch’s theorem has been extended to cover generalized mass-action kinetics (Müller et al. 2019; Craciun et al. 2019).

### Example

We consider a parametrized system of two non-overlapping trinomials in two variables, 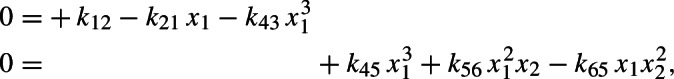 arising from a reaction network.^1^ Equivalently,

$$\begin{aligned} A \left( c\circ x^B \right) = 0, \end{aligned}$$

where

$$\begin{aligned} A= \begin{pmatrix} 1 & -1 & -1 & 0 & 0 & 0 \\ 0 & 0 & 0 & 1 & 1 & -1 \end{pmatrix}, \\ B = \begin{pmatrix} 0 & 1 & 3 & 3 & 2 & 1 \\ 0 & 0 & 0 & 0 & 1 & 2 \end{pmatrix}, \\ c = (k_{12}, k_{21}, k_{43}, k_{45}, k_{56}, k_{65})^\textsf{T}. \end{aligned}$$

Clearly, the coefficient matrix $$A = \begin{pmatrix} A_1&A_2 \end{pmatrix} \in \mathbb {R}^{2 \times 6}$$ has two blocks $A_1, A_2 \in \mathbb {R}^{2 \times 3}$ such that $\ker A = \ker A_1 \times \ker A_2$. Accordingly, the exponent matrix $$B = \begin{pmatrix} B_1&B_2 \end{pmatrix}$$ has two blocks

$$ B_1 = \begin{pmatrix} 0 & 1 & 3\\ 0 & 0 & 0 \end{pmatrix} \quad \text {and} \quad B_2 = \begin{pmatrix} 3 & 2 & 1 \\ 0 & 1 & 2 \end{pmatrix}, $$

that is, there are two *classes* of monomials, 1, $x_1$, $x_1^3$ and $x_1^3$, $x_1^2 x_2$, $x_1 x_2^2$, respectively. Further, $$c = \left( {\begin{array}{c}c_1\\ c_2\end{array}}\right) $$ with $c_1 = (k_{12}, k_{21}, k_{43})^\textsf{T}$ and $c_2 = (k_{45}, k_{56}, k_{65})^\textsf{T}$.

For the coefficient cone $C = \ker A \cap \mathbb {R}^6_>$, we find $C = C_1 \times C_2$ with $C_j = \ker A_j \cap \mathbb {R}^3_>$, and for the coefficient polytope $P = C \cap \Delta$, where $\Delta = \Delta ^2 \times \Delta ^2$ is a product of the standard simplices $\Delta ^2 = \{ y \in \mathbb {R}^3_\ge \mid 1_3 \cdot y = 1 \}$, we find

$$\begin{aligned} P = P_1 \times P_2 \end{aligned}$$

with $$P_j = \ker A_j \cap \mathbb {R}^3_> \cap \Delta ^2$$ and $$\dim P_1 = \dim P_2 = 1$$.

By appending a Cayley matrix (rows of ones corresponding to the two blocks) to the exponent matrix *B*, we obtain

$$ \mathcal {B} = \begin{pmatrix} 0 & 1 & 3 & 3 & 2 & 1 \\ 0 & 0 & 0 & 0 & 1 & 2 \\ 1 & 1 & 1 & 0 & 0 & 0 \\ 0 & 0 & 0 & 1 & 1 & 1 \end{pmatrix}, $$

which allows to determine the monomial dependency subspace $D = \ker \mathcal {B}$ and the dependency $d = \dim D$. In particular, $d = \dim (D) = 2$. In this example, the polynomial system is *decomposable*, that is, $D = D_1 \times D_2$ with $$D_1 = {{\,\textrm{im}\,}}\begin{pmatrix} 2&-3&1 \end{pmatrix}^\textsf{T}$$ and $$D_2 = {{\,\textrm{im}\,}}\begin{pmatrix} 1&-2&1 \end{pmatrix}^\textsf{T}$$ corresponding to the decomposition $\ker A = \ker A_1 \times \ker A_2$. Clearly, $d_1 = \dim D_1 = d_2 = \dim D_2 = 1$ and hence $d = d_1 + d_2$.

Finally, we obtain the monomial difference matrix *M* from $$B = \begin{pmatrix} B_1&B_2 \end{pmatrix}$$ by taking the differences of the first two columns and the last column within the two blocks,

$$ M = \begin{pmatrix} -3 & -2 & 2 & 1 \\ 0 & 0 & -2 & -1 \end{pmatrix}. $$

This allows to determine the monomial difference subspace $L = {{\,\textrm{im}\,}}M$. In fact, $L = \mathbb {R}^2$.

## Dependency One Systems

First, we consider systems with one class, second, we consider decomposable systems.

### One Class

For one class, we consider $d=\dim P =1$. (The case $d=\dim P =0$ is trivial.)

#### Definition 4

For a parametrized system of generalized polynomial equations $A \, ( c \circ x^B ) = 0$ with $\ker A \cap \mathbb {R}^m_> \ne \emptyset$, one class, one-dimensional coefficient polytope, and monomial dependency one, let $y^1, y^2 \in {(\ker A \cap \mathbb {R}^m_\ge )}$ be the two vertices of the coefficient polytope, let $q = (y^1 - y^2) \circ (y^1 + y^2)^{-1} \in \mathbb {R}^m$, and assume that $1 = q_1 \ge \cdots \ge q_m = -1$ (after reordering of the index set $\{1,\ldots ,m\}$). Further, let $I_1, \ldots , I_\omega \subset \{1, \ldots ,m\}$ be $\omega$ equivalence classes corresponding to equal (consecutive) components of *q*, and let $\bar{q} \in \mathbb {R}^\omega$ with $\bar{q}_i = q_{i'}$ for $i' \in I_i$ be the vector of different *q*’s. Finally, let $b \in \mathbb {R}^m$ with ${{\,\textrm{im}\,}}b = \ker (\mathcal {B})$, and let $\bar{b} \in \mathbb {R}^\omega$ with $\bar{b}_i = \sum _{i' \in I_i} b_{i'}$ be the vector of lumped *b*’s.

#### Theorem 5

($d=1$, one class) Let $A \, ( c \circ x^B ) = 0$ be a parametrized system of generalized polynomial equations with $\ker A \cap \mathbb {R}^m_> \ne \emptyset$, one class, one-dimensional coefficient polytope ($\dim P = 1$), and monomial dependency one ($d=1$). Then, $|Y_c| = 1$ for all *c* if

$\displaystyle \sum _{i'=1}^{i} \bar{b}_{i'} \ge 0$ for all $i=1,\ldots ,\omega -1$ (or “$\le 0$” for all *i*) and $\bar{b}_1 \cdot \bar{b}_\omega < 0$.

#### Proof

Let $\hat{y} = \frac{y^1 - y^2}{2}$, $\bar{y} = \frac{y^1 + y^2}{2} > 0$, and hence $q = \hat{y}\circ {\bar{y}}^{-1}$. Every $y \in P$ can be written as $y = \bar{y} + t \hat{y}$ with $t \in (-1,1)$, and the binomial condition $y^{b} = c^b$ for $y \in P$ can be written as $(\bar{y} + t \hat{y})^b = c^b$ for $t \in (-1,1)$ or, after division by $\bar{y}$, as $f(t):= (1 + t q)^b = c^b \, \bar{y}^{-b} =: c^*$. After considering equal components of *q*,

$$ f(t) = \prod _{i=1}^m (1+tq_i)^{b_i} = \prod _{i=1}^{\omega } (1+t\bar{q}_i)^{\bar{b}_i}. $$

Now, let $\bar{b}_1 \cdot \bar{b}_\omega < 0$, in particular, $\bar{b}_1 > 0$ and $\bar{b}_\omega < 0$. (The other case is analogous.) Clearly, $\bar{q}_1 = 1$ implies $f(-1) \rightarrow 0$, and $\bar{q}_\omega = -1$ implies $f(1) \rightarrow \infty$. By continuity, there is a solution to $f(t) = c^*$ for all $c^*$ and hence for all *c*. That is, $|Y_c|\ge 1$ for all *c*.

Moreover, $$f'(t) = f(t) \, h(t)$$ with

$$ h(t) = \sum _{i=1}^\omega \bar{b}_i \, \frac{\bar{q}_i}{1 + t \bar{q}_i} = \sum _{i=1}^{\omega -1} \left( \sum _{i'=1}^i \bar{b}_{i'} \right) \underbrace{\left( \frac{\bar{q}_i}{1 + t \bar{q}_i} - \frac{\bar{q}_{i+1}}{1 + t \bar{q}_{i+1}} \right) }_{>0}. $$

Now, also let $\sum _{i'=1}^{i} \bar{b}_{i'} \ge 0$ for all $i=1,\ldots ,\omega -1$ (or “$\le 0$” for all *i*). Altogether, this implies $f'(t)> 0$ (or $f'(t)< 0$). That is, $|Y_c| \le 1$ for all *c*. $\square$

Recently, existence of a unique, nondegenerate solution to a parametrized system of generalized polynomial equations (with arbitrary dependency and using one class) for all parameters) has been characterized using Hadamard’s Global Inversion Theorem (Deshpande and Müller 2024).

Notably, existence (without uniqueness) of a solution on the coefficient polytope for all parameters can be characterized.

#### Theorem 6

Let $A \, ( c \circ x^B ) = 0$ be a parametrized system of generalized polynomial equations with $\ker A \cap \mathbb {R}^m_> \ne \emptyset$, one class, one-dimensional coefficient polytope, and monomial dependency one. The following statements are equivalent:

1. $$|Y_c| \ge 1$$ for all *c*.
2. $$\bar{b}_1\cdot \bar{b}_\omega <0$$.

#### Proof

See Appendix A. $$\square $$

### Decomposable Systems

#### Definition 7

For a parametrized system of generalized polynomial equations $A \, (c \circ x^B) = 0$ with $\ker A \cap \mathbb {R}^m_> \ne \emptyset$ and $\ell$ classes, let $P_j$ be the coefficient polytope and $d_j$ be the monomial dependency of the subsystem $A_j \, (c^j \circ x^{B_j}) = 0$, $j=1,\ldots ,\ell$. If $\dim P_j=1$, let $y^{j,1}, y^{j,2} \in {(\ker A_j \cap \mathbb {R}^{m_j}_\ge )}$ be the two vertices of $P_j$, let $q^j = (y^{j,1} - y^{j,2}) \circ (y^{j,1} + y^{j,2})^{-1} \in \mathbb {R}^{m_j}$, and assume that $1 = q^j_1 \ge q^j_2 \ge \cdots \ge q^j_{m_j} = -1$ (after reordering of the index set $\{1,\ldots ,m_j\}$). Further, let $I^j_1, I^j_2, \ldots , I^j_{\omega _j} \subset \{1, \ldots , m_j\}$ be $\omega _j$ equivalence classes corresponding to equal (consecutive) components of $q^j$. If $d_j=1$, let $b^j \in \mathbb {R}^{m_j}$ with ${{\,\textrm{im}\,}}b^j = \ker (\mathcal {B}_j)$, and let $\bar{b}^j \in \mathbb {R}^{\omega _j}$ with $\bar{b}^j_i = \sum _{i' \in I_i} b^j_{i'}$ be the vector of lumped $b^j$’s.

#### Theorem 8

($d_j \le 1$) Let $A \, (c \circ x^B) = 0$ be a parametrized system of generalized polynomial equations with $\ell$ classes that fulfills the following conditions:

(i) $$\ker A \cap \mathbb {R}^m_> \ne \emptyset $$.
(ii) $$d = d_1 + \cdots + d_{\ell }$$.
(iii) For every (class) $j=1,\ldots ,\ell$,
  $$d_j = \dim P_j \le 1$$.
  If $$d_j=1$$, then
    $\displaystyle \sum _{i'=1}^{i} \bar{b}^j_{i'} \ge 0$ for all $i=1,\ldots ,\omega _j-1$ (or “$\le 0$” for all *i*)
    $$\bar{b}^j_1 \cdot \bar{b}^j_{\omega _j} < 0$$.

Then, $|Y_c| = 1$ for all *c*.

#### Proof

By (i), the coefficient polytope $P = P_1 \times \cdots \times P_\ell$ is non-empty. By (ii) and (Müller and Regensburger 2026, Section 3.2), the system is *decomposable*. By (Müller and Regensburger 2026, Proposition 10), $Y_c = Y_{c,1} \times \cdots \times Y_{c,{\ell }}$, that is, the solution set on the coefficient polytope is a direct product. In particular, $|Y_c| = |Y_{c,1}| \cdot \, \cdots \, \cdot |Y_{c,\ell }|$. By the first item in (iii), $d_j = \dim P_j \le 1$.

Case $d_j = \dim P_j = 0$: $P_j$ is a point, and there is no binomial condition on $P_j$. Hence, $|Y_{c,j}|=1$.

Case $d_j = \dim P_j = 1$: By the second item in (iii), $\sum _{i'=1}^{i} \bar{b}^j_{i'} \ge 0$ for all $i=1,\ldots ,\omega _j-1$ (or “$\le 0$” for all *i*) and $\bar{b}^j_1 \cdot \bar{b}^j_{\omega _j} < 0$. By Theorem 5 for the subsystem $A_j \, (c^j \circ x^{B_j}) = 0$, $|Y_{c,j}|=1$.

Altogether, $|Y_c| = |Y_{c,1}| \cdot \, \cdots \, \cdot |Y_{c,\ell }| = 1$, and all implications hold for all *c*. $\square$

## Reaction Networks

We recall basic notions for reaction networks with mass-action kinetics (Adleman et al. 2014, Voit et al. 2015, Yu and Craciun 2018, Gunawardena 2003) following (Müller and Regensburger 2014). Note that we use index notation (rather than pure cardinality notation) in the definition of matrices, thereby avoiding arbitrary orderings of the underlying sets.

A reaction network (*G*, *y*) is given by a simple directed graph $G=(V,E)$ with a finite set of vertices *V* and edge set $E \subseteq V \times V$ together with an injective map ${y :V \rightarrow \mathbb {R}^n}$ (a matrix $Y \in \mathbb {R}^{n \times V}$). Every vertex $i \in V$ is labeled with a *(stoichiometric) complex* $y(i) \in \mathbb {R}^n$, and every edge $(i \rightarrow i') \in E$ represents a *reaction* ${y(i) \rightarrow y(i')}$. The restriction of the map *y* (the matrix *Y*) to the set of source vertices $V_s \subseteq V$ is denoted by $y_s :V_s \rightarrow \mathbb {R}^n$ (respectively, $Y_s \in \mathbb {R}^{n \times V_s}$). If all components of the graph are strongly connected, the network is called *weakly reversible*.

### Remark

In the classical definition of a reaction network, complexes (like reactions) are primary objects and correspond to the vertices (and edges) of the induced complex-reaction graph. Such reaction networks can also be represented as Euclidean-embedded graphs (Craciun 2015). See also (Craciun 2019; Craciun and Deshpande 2020).

A *mass-action system* $(G_k,y)$ is given by a reaction network (*G*, *y*) and positive edge labels $k \in \mathbb {R}^E_>$. Every edge/reaction $(i \rightarrow i') \in E$ is labeled with a *rate constant* $k_{i \rightarrow i'} > 0$.

The associated ODE system for the non-negative *concentrations* $x \in \mathbb {R}^n_\ge$ (of *n* chemical species) is given by

1 $$\begin{aligned} \frac{\text {d} x}{\text {d} t} = \sum _{(i \rightarrow i') \in E} k_{i \rightarrow i'} \, x^{y(i)} \big ( y(i')-y(i) \big ) . \end{aligned}$$

The sum ranges over all reactions, and every summand is a product of the *reaction rate* $k_{i \rightarrow i'} \, x^{y(i)}$, involving a monomial $x^{y} = \prod _{j=1}^n (x_j)^{y_j}$ determined by the stoichiometric complex of the reactant, and the *reaction vector* $y(i')-y(i)$ given by the stoichiometric complexes of product and reactant.

Let $I_E \in \{-1,0,1\}^{V \times E}$ and $I_{E,s} \in \{0,1\}^{V_s \times E}$ be the incidence and source matrices of the digraph *G*, respectively, and $R_k = I_E {{\,\textrm{diag}\,}}(k) (I_{E,s})^\textsf{T}\in \mathbb {R}^{V \times V_s}$ be the rectangular “Laplacian matrix”. (For details on the index notation, see Appendix B.1.) Further, recall the (source) complex matrices, $Y \in \mathbb {R}^{n \times V}$ and $Y_s \in \mathbb {R}^{n \times V_s}$, and let $N = Y I_E \in \mathbb {R}^{n \times E}$ be the stoichiometric matrix. Then, the right-hand-side of (1) can be written in matrix form and decomposed into stoichiometric and graphical contributions,

2 $$\begin{aligned} \begin{aligned} \frac{\text {d} x}{\text {d} t}&= \underbrace{Y I_E}_N \left( k \circ x^{Y_s I_{E,s}} \right) = Y \underbrace{I_E {{\,\textrm{diag}\,}}(k) (I_{E,s})^\textsf{T}}_{R_k} x^{Y_s} \\&= \Gamma _k \, x^{Y_s} . \end{aligned} \end{aligned}$$

In the last step, we have introduced the *kinetic matrix* $\Gamma _k = Y R_k \in \mathbb {R}^{n \times V_s}$, in analogy to the stoichiometric matrix $N = Y I_E$.

### Remark

Traditionally, one uses the source matrix $I'_{E,s} \in \{0,1\}^{V \times E}$ which involves all vertices (not just the source vertices), and one obtains

$$ \frac{\text {d} x}{\text {d} t} = Y I_E \left( k \circ x^{Y I'_{E,s}} \right) = Y \underbrace{I_E {{\,\textrm{diag}\,}}(k) (I'_{E,s})^\textsf{T}}_{\mathcal {L}_k} x^{Y} $$

with the (square) Laplacian matrix $\mathcal {L}_k \in \mathbb {R}^{V \times V}$. This formulation can be misleading since columns of $\mathcal {L}_k$ corresponding to non-source vertices are zero, and, after multiplication, non-source monomials do not appear in $\frac{\text {d} x}{\text {d} t} = Y\mathcal {L}_k \, x^{Y}$. Indeed, $R_k$ arises from $\mathcal {L}_k$ by deleting zero columns, and ${{\,\textrm{im}\,}}R_k = {{\,\textrm{im}\,}}\mathcal {L}_k$.

The change over time lies in the *kinetic subspace* $K = {{\,\textrm{im}\,}}(Y R_k)$ and further in the stoichiometric subspace *stoichiometric subspace* $S = {{\,\textrm{im}\,}}(Y I_E)$,

$$ \frac{\text {d} x}{\text {d} t} \in K \subseteq S. $$

Hence, trajectories are confined to cosets of *K* and *S*, respectively, that is, $x(t) \in x(0)+K \subseteq x(0) + S$. For positive $x' \in \mathbb {R}^n_>$, the sets $(x'+K) \subseteq (x'+S) \cap \mathbb {R}^n_>$ are called *kinetic* and *stoichiometric compatibility classes*, respectively.

Finally, we introduce non-negative integer characteristics of a graph or a reaction network. In particular, let *l* be the number of *components* of *G* (not to be confused with $\ell$ defined below), let *t* be the number of *absorbing strong components*, and let $t'$ be the number of non-singleton absorbing strong components. It is well known that

$$ \dim (\ker I_E^\textsf{T}) = l \quad \text {and} \quad \dim (\ker \mathcal {L}_k) = t, $$

which further implies

$$ \dim ({{\,\textrm{im}\,}}I_E) = |V| - l \quad \text {and} \quad \dim ({{\,\textrm{im}\,}}R_k) = \dim ({{\,\textrm{im}\,}}\mathcal {L}_k) = |V| - t. $$

Analogously,

$$ \dim (\ker R_k) = t'. $$

Most importantly, the (stoichiometric) *deficiency* is given by

$$ \delta = \dim (\ker Y \cap {{\,\textrm{im}\,}}I_E) = |V| - l - \dim (S). $$

From the facts above, it can be easily shown that

$$ t=l \quad \implies \quad K=S \quad \implies \quad \delta \ge t-l. $$

Mass-action systems with $K \ne S$ can be “pathological”. The assumption $t=l$ in the classical deficiency one theorem (Feinberg 1987) rules out such systems. In the extended deficiency one theorem (Feinberg 1995a) (for independent subnetworks), this assumption is missing.

For a summary of the notation introduced in this section, see Table 1(a). For an illustration, we continue the example from Sect. 2.

### Example

*(continued)* We return to the reaction network (with rate constants)or, equivalently, the “embedded” graph 
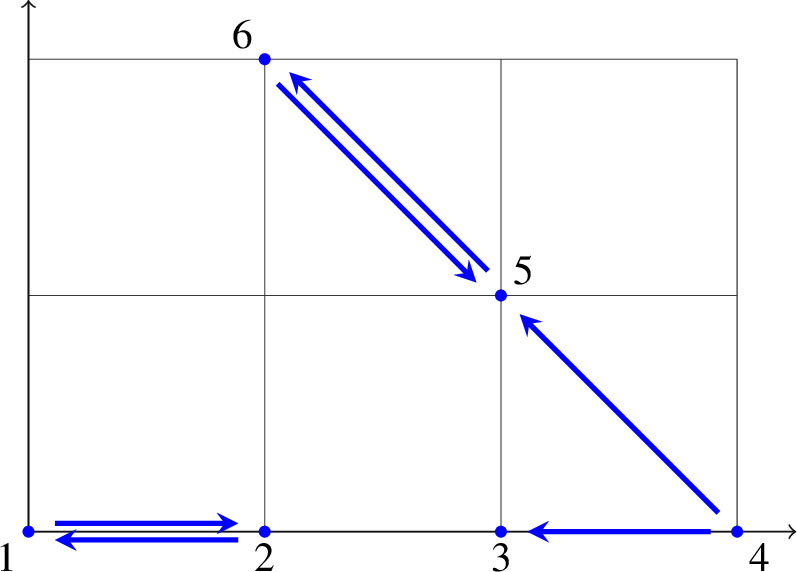
 In our definition, the reaction network (*G*, *y*) is given by the graph $G=(V,E)$ with vertex set $V=\{1,2,3,4,5,6\}$ and edge set $E=\{12,21,43,45,56,65\}$ (where we write *ij* short for $i\rightarrow j$) and the map $y :V \rightarrow \mathbb {R}^n$ with $n=2$ and $$y(1)=\left( {\begin{array}{c}0\\ 0\end{array}}\right) , y(2) = \left( {\begin{array}{c}1\\ 0\end{array}}\right) $$, $$y(3)=\left( {\begin{array}{c}2\\ 0\end{array}}\right) , y(4) = \left( {\begin{array}{c}3\\ 0\end{array}}\right) $$, $$y(5)=\left( {\begin{array}{c}2\\ 1\end{array}}\right) , y(6) = \left( {\begin{array}{c}1\\ 2\end{array}}\right) $$ (which labels vertices with complexes). The mass-action system $(G_k,y)$ further labels edges with positive rate constants $k = (k_{12}, k_{21}, k_{43}, k_{45}, k_{56}, k_{65})^\textsf{T}\in \mathbb {R}^E_>$. Note that the set of source vertices is $V_s=\{1,2,4,5,6\}$.

First, the $V \times E$ incidence and $V_s \times E$ source matrices (of the graph) are given bywith resulting $V \times V_s$ (rectangular) Laplacian matrixSecond, the $n \times V$ complex and $n \times V_s$ source complex matrices (arising from the underlying map) are given by

$$\begin{aligned}. \end{aligned}$$

Third, the $n \times E$ stoichiometric and $n \times V_s$ kinetic matrices are given byandThey define the stoichiometric and kinetic subspaces, $S = {{\,\textrm{im}\,}}N = \mathbb {R}^2$ and $K= {{\,\textrm{im}\,}}\Gamma _k = \mathbb {R}^2$ (for all *k*).

The network has $l=2$ components (with vertex sets $\{1,2\}$ and $\{3,4,5,6\}$) and $t=3$ absorbing strong components (with vertex sets $\{1,2\}$, $\{3\}$, and $\{5,6\}$), in particular, $t\ne l$. The resulting deficiency is $\delta = |V|-l-\dim S = 6 - 2 - 2 = 2$. Further, we note that the deficiencies of the two components are $\delta _1 = 2 - 1 - 1 = 0$ and $\delta _2 = 4 - 1 - 2 = 1$, and hence $\delta \ne \delta _1+\delta _2$.

## Network Decomposition

First, we decompose a reaction network/mass-action system and the associated ODE. Then, in Subsection 5.1, we formulate the resulting polynomial equations for positive equilibria. Only in Subsection 5.2, we assume independent subnetworks (in the sense of Feinberg (1987)) and address the decomposability of the polynomial equations (in the sense of Müller and Regensburger (2026)).

In order to decompose the right-hand side of the ODE of a mass-action system,

3 $$\begin{aligned} \frac{\text {d} x}{\text {d} t} = Y I_E \left( k \circ x^{Y_s I_{E,s}} \right) , \end{aligned}$$

we proceed in three steps.

(i) We assume that the edge set is partitioned into $\ell$ disjoint subsets, $E = E^1 \dot{\cup }\cdots \dot{\cup }E^\ell$. (For the moment, the partition is arbitrary. In Subsection 5.2, it will arise from writing the kernel of $N = Y I_E$ as a direct product.) The partition induces the subgraphs $G^j = (V^j,E^j)$, $j = 1, \ldots , \ell$, with vertex sets $V^j$, source vertex sets $V_s^j$, and non-source vertex sets $V_\textit{ns}^j$. Note that the vertex sets need not be disjoint. Further note that absorbing strong components are either non-source vertices or non-singleton strong components. Let $t_j$ denote the number of absorbing strong components (of the subgraph $G^j$) and $t'_j$ denote the number of non-singleton absorbing strong components. Clearly, $t_j = t'_j + |V^j_\textit{ns}|$. The subgraph $G^j$ has incidence and source matrices

$$ I_E^j \in \{-1,0,1\}^{V^j \times E^j}, \quad I_{E,s}^j \in \{0,1\}^{V_s^j \times E^j}. $$

The corresponding subnetwork $(G^j,y^j)$ has complex matrix $Y^j \in \mathbb {R}^{n \times V^j}$ and source complex matrix $Y_s^j \in \mathbb {R}^{n \times V_s^j}$. (All matrices for a subgraph are submatrices of the corresponding matrices for the full graph.) Clearly,

$$ Y I_E = \begin{pmatrix} Y^1 I_E^1&\ldots&Y^\ell I_E^\ell \end{pmatrix} \quad \text {and} \quad Y_s \, I_{E,s} = \begin{pmatrix} Y_s^1 I_{E,s}^1&\ldots&Y_s^\ell I_{E,s}^\ell \end{pmatrix}. $$

The vector of parameters $k \in \mathbb {R}^E_>$ is partitioned accordingly,

$$ k = \begin{pmatrix} k^1 \\ \vdots \\ k^\ell \end{pmatrix} $$

with $k^j \in \mathbb {R}_>^{E^j}$, and the rectangular Laplacian matrix of the mass-action system $(G_k^j,y^j)$ is given by

$$ R_k^j = I_E^j {{\,\textrm{diag}\,}}(k^j) (I_{E,s}^j)^\textsf{T}\in \mathbb {R}^{V^j \times V_s^j}. $$

(ii) In turn, we define “combined” block(-diagonal) matrices from the matrices of the subgraphs. Since the vertex sets need not be disjoint, we form the *disjoint unions* of (source) vertex sets, $V^\sqcup = V^1 \sqcup \cdots \sqcup V^\ell$ and $V_s^\sqcup = V_s^1 \sqcup \cdots \sqcup V_s^\ell$. We introduce the combined incidence and source matrices

$$ I^*_E = \begin{pmatrix} I_E^1 & & 0 \\ & \ddots & \\ 0 & & I_E^\ell \end{pmatrix} \in \{-1,0,1\}^{V^\sqcup \times E}, \quad I^*_{E,s} = \begin{pmatrix} I_{E,s}^1 & & 0 \\ & \ddots & \\ 0 & & I_{E,s}^\ell \end{pmatrix} \in \{0,1\}^{V_s^\sqcup \times E} $$

and the combined rectangular Laplacian matrix

$$ R^*_k = I^*_E {{\,\textrm{diag}\,}}(k) (I^*_{E,s})^\textsf{T}= \begin{pmatrix} R_k^1 & & 0 \\ & \ddots & \\ 0 & & R_k^\ell \end{pmatrix} \in \mathbb {R}^{V^\sqcup \times V_s^\sqcup }. $$

The (source) complex matrices can be combined accordingly,

$$ Y^* = \begin{pmatrix} Y^1&\ldots&Y^\ell \end{pmatrix} \in \mathbb {R}^{n \times V^\sqcup }, \quad Y_s^* = \begin{pmatrix} Y_s^1&\ldots&Y_s^\ell \end{pmatrix} \in \mathbb {R}^{n \times V_s^\sqcup }. $$

On the one hand, $Y^* I^*_E = Y I_E.$ On the other hand, $Y_s^* = Y_s \, I^*_{V,s}$ with a matrix $I^*_{V,s} \in \{0,1\}^{V_s \times V_s^\sqcup }$ that assigns to every source vertex of a subgraph the corresponding source vertex of the full graph.

(iii) Now, we return to the ODE (3). Like the matrix $Y I_E$, the vector of monomials ${x^{Y_s I_{E,s}} \in \mathbb {R}^E_\ge }$ has $\ell$ blocks and can be decomposed as

$$\begin{aligned} x^{Y_s I_{E,s}}&= \begin{pmatrix} x^{Y_s^1 I_{E,s}^1} \\ \vdots \\ x^{Y_s^\ell I_{E,s}^\ell } \end{pmatrix} = \begin{pmatrix} (I_{E,s}^1)^\textsf{T}x^{Y_s^1} \\ \vdots \\ (I_{E,s}^\ell )^\textsf{T}x^{Y_s^\ell } \end{pmatrix} \\&= \begin{pmatrix} (I_{E,s}^1)^\textsf{T}& & 0 \\ & \ddots & \\ 0 & & (I_{E,s}^\ell )^\textsf{T}\end{pmatrix} \begin{pmatrix} x^{Y_s^1} \\ \vdots \\ x^{Y_s^\ell } \end{pmatrix} = (I^*_{E,s})^\textsf{T}x^{Y^*_s} . \end{aligned}$$

Using the definitions above, we decompose the right-hand side of the ODE as

$$\begin{aligned} \frac{\text {d} x}{\text {d} t}&= Y I_E \left( k \circ x^{Y_s I_{E,s}} \right) = Y I_E {{\,\textrm{diag}\,}}(k) \, x^{Y_s I_{E,s}} \\&= Y^* I^*_E {{\,\textrm{diag}\,}}(k) (I^*_{E,s})^\textsf{T}x^{Y^*_s} = Y^* R^*_k \, x^{Y^*_s} . \end{aligned}$$

Finally, we introduce the kinetic matrices

$$ \Gamma _k^j = Y^j R_k^j \in \mathbb {R}^{n \times V_s^j}, \quad j=1,\ldots ,\ell , $$

and the combined kinetic matrix

$$ \Gamma _k^* = Y^* R_k^* = \begin{pmatrix} \Gamma _k^1&\ldots&\Gamma _k^\ell \end{pmatrix} = \begin{pmatrix} Y^1 R_k^1&\ldots&Y^\ell R_k^\ell \end{pmatrix} \in \mathbb {R}^{n \times V_s^\sqcup } $$

and summarize the decomposition as

4 $$\begin{aligned} \frac{\text {d} x}{\text {d} t} = \Gamma _k^* \, x^{Y^*_s} = \sum _{j =1}^\ell \Gamma _k^j \, x^{Y_s^j}. \end{aligned}$$

Before we proceed with the treatment of the resulting polynomial equations, we introduce linear subspaces associated with a subnetwork $(G^j,y^j)$ or a mass-action system $(G^j_k,y^j)$, $j=1,\ldots ,\ell$. As above, we define the stoichiometric and kinetic subspaces $S_j = {{\,\textrm{im}\,}}(Y^j I_E^j)$ and $K_j = {{\,\textrm{im}\,}}(Y^j R^j_k)$, where $K_j \subseteq S_j$ since ${{\,\textrm{im}\,}}R^j_k \subseteq {{\,\textrm{im}\,}}I_E^j$, and further the combined kinetic subspace $K^* = {{\,\textrm{im}\,}}(Y^* R_k^*) = \sum _j K_j$. We record the following fact.

### Fact 9

Let (*G*, *y*) be a reaction network decomposed into $\ell$ subnetworks. Then, $S = \sum _{j=1}^\ell S_j$ and $K \subseteq K^* = \sum _{j=1}^\ell K_j$.

### Proof

Recall $$Y I_E = Y^* I^*_E = \begin{pmatrix} Y^1 I_E^1&\ldots&Y^\ell I_E^\ell \end{pmatrix} $$ and $$Y^* R_k^* = \begin{pmatrix} Y^1 R_k^1&\ldots&Y^\ell R_k^\ell \end{pmatrix}$$. First,

$$ S = {{\,\textrm{im}\,}}(Y I_E) = {{\,\textrm{im}\,}}(Y^1 I_E^1) + \cdots + {{\,\textrm{im}\,}}(Y^\ell I_E^\ell ) = S^1 + \cdots + S^\ell . $$

Second, $K = {{\,\textrm{im}\,}}(Y R_k)$ and $K^* = {{\,\textrm{im}\,}}(Y^* R_k^*)$, where

$$ Y R_k = Y I_E {{\,\textrm{diag}\,}}(k) (I_{E,s})^\textsf{T}$$

and

$$ Y^* R_k^* = Y^* I^*_E {{\,\textrm{diag}\,}}(k) (I^*_{E,s})^\textsf{T}. $$

Since $Y I_E = Y^* I^*_E$ and ${{\,\textrm{im}\,}}(I_{E,s})^\textsf{T}\subseteq {{\,\textrm{im}\,}}(I^*_{E,s})^\textsf{T}$, we have ${{\,\textrm{im}\,}}(Y R_k) \subseteq {{\,\textrm{im}\,}}(Y^* R_k^*)$. $\square$

### Positive Equilibria

Finally, we consider positive equilibria of the ODE (3), that is, $x \in \mathbb {R}_>^n$ such that $\frac{\text {d} x}{\text {d} t} = 0$. Clearly, we can specify them in the form $A \, (c \circ x^B) = 0$ as

5a $$\begin{aligned} N \, (k \circ x^{Y_s I_{E,s}}) = 0 , \end{aligned}$$

that is, with $A = N \in \mathbb {R}^{n \times E}$, $c = k \in \mathbb {R}_>^E$, $B = Y_s \, I_{E,s} \in \mathbb {R}^{n \times E}$; equivalently, using the decomposition (4), we can specify them as

5b $$\begin{aligned} \Gamma _k^* \, x^{Y^*_s} = 0 , \end{aligned}$$

that is, with $A = \Gamma _k^* \in \mathbb {R}^{n \times V_s^\sqcup }$, $c = 1$, and $B = Y^*_s \in \mathbb {R}^{n \times V_s^\sqcup }$.

In Equation (5a), the coefficient matrix $A=N$ (and hence the classes determined by its kernel) do not depend on the parameters *k*. However, there may be repeated monomials (within classes) giving rise to trivial dependencies. In Equation (5b), the coefficient matrix $A=\Gamma _k^*$ does depend on *k*, but repeated monomials are handled via the (rectangular) Laplacian matrix (which also eliminates non-source monomials). Hence, we will consider Equation (5b), but use classes arising from Equation (5a).

### Independent Subnetworks and Decomposability

Let (*G*, *y*) with $G=(V,E)$ be a reaction network. In the following, we assume that the partition of the edge set, $E = E^1 \dot{\cup }\cdots \dot{\cup }E^\ell$, arises from a decomposition of $\ker N$ (as a *direct product*) or, equivalently, from a decomposition of $S={{\,\textrm{im}\,}}N$ (as a *direct sum*). That is,

$$ N = Y I_E = \begin{pmatrix} N^1&\ldots&N^\ell \end{pmatrix} = \begin{pmatrix} Y^1 I_E^1&\ldots&Y^\ell I_E^\ell \end{pmatrix} $$

such that

6a $$\begin{aligned} \ker N = \ker N^1 \times \cdots \times \ker N^\ell . \end{aligned}$$

Equivalently,

$$ S = S_1 \oplus \cdots \oplus S_\ell . $$

In the terminology of (Feinberg 1987), the resulting subnetworks $(G^j,y^j)$ are *independent*. In the setting of the deficiency one theorem, we will assume that the subgraphs $G^j$ are connected (and say that the subnetworks are connected), see Section 7.

Now, let $(G_k,y)$ be the corresponding mass-action system with combined kinetic matrix

$$ \Gamma _k^* = Y^* R_k^* = \begin{pmatrix} \Gamma _k^1&\ldots&\Gamma _k^\ell \end{pmatrix} = \begin{pmatrix} Y^1 R_k^1&\ldots&Y^\ell R_k^\ell \end{pmatrix} . $$

By Proposition 10 below,

6b $$\begin{aligned} \ker \Gamma _k^* = \ker \Gamma _k^1 \times \cdots \times \ker \Gamma _k^\ell . \end{aligned}$$

Equivalently,

$$ K^* = K_1 \oplus \cdots \oplus K_\ell . $$

In the terminology of Müller and Regensburger (2026) (for the polynomial equations $\Gamma _k^* \, x^{Y_s^*} = 0$), the decomposition of $\ker \Gamma _k^* \subseteq \mathbb {R}^{ V_s^\sqcup }$ induces a partition of the source vertex set $V_s^\sqcup = V_s^1 \sqcup \cdots \sqcup V_s^\ell$ into the $\ell$ *classes* $V_s^j$. It remains to provide a formal argument.

#### Proposition 10

Let (*G*, *y*) be a reaction network decomposed into $\ell$ independent subnetworks and $(G_k,y)$ be the corresponding mass-action system. Then, (6a) implies (6b).

#### Proof

Consider $\xi \in \mathbb {R}^{V_s^\sqcup }$ with blocks $\xi ^j \in \mathbb {R}^{V_s^j}$, $j=1,\ldots ,\ell$, and assume $\Gamma _k^* \, \xi = \sum _{j=1}^\ell \Gamma _k^j \, \xi ^j = 0$. Further, introduce $\alpha ^j = {{\,\textrm{diag}\,}}(k^j) (I_{E,s}^j)^\textsf{T}\xi ^j \in \mathbb {R}^{E^j}$. Then,

$$ N^j \alpha ^j = Y^j I_E^j {{\,\textrm{diag}\,}}(k^j) (I_{E,s}^j)^\textsf{T}\xi ^j = Y^j R_k^j \, \xi ^j = \Gamma _k^j \, \xi ^j $$

and hence $\sum _{j=1}^\ell N^j \alpha ^j = 0$. By (6a), $N^j \alpha ^j = \Gamma _k^j \, \xi ^j = 0$. That is, (6b). $\square$

To summarize, the decomposition of the kernel of the stoichiometric matrix implies a partition of the edge set (and a corresponding decomposition of the network into *independent subnetworks*) and further a decomposition of the kernel of the combined kinetic matrix (and a corresponding partition of the source vertex set into *classes*). An algorithm for network decomposition has been developed in Hernandez and la Cruz (2021) and applied to derive steady states analytically in (Hernandez et al. 2023).

For the polynomial equations $\Gamma _k^* \, x^{Y_s^*} = 0$, the monomial dependency and difference subspaces, *D* and *L*, are given in Definition 1 (with $B=Y_s^*$). Assuming $\ell$ classes, we introduce corresponding subspaces $D_j$ and $L_j$, $j = 1,\ldots ,\ell$. In particular, $L_j = {{\,\textrm{im}\,}}(Y_s^j I_{\mathcal {E}_s}^j)$, where $I_{\mathcal {E}_s}^j \in \{-1,0,1\}^{V_s^j \times \mathcal {E}_s}$ is the incidence matrix of a star-shaped graph $(V_s^j,\mathcal {E}_s)$ (on the source vertices) with $|\mathcal {E}_s| = |V_s^j| - 1$.

As defined in (Müller and Regensburger 2026, Section 3.2), the polynomial equations $\Gamma _k^* \, x^{Y_s^*} = 0$ are *decomposable* if *D* has the decomposition $D=D_1 \times \cdots \times D_\ell$, corresponding to the decomposition of $\ker \Gamma _k^*$; equivalently, $d = d_1 + \cdots + d_{\ell }$, where $d_j = \dim D_j$. We provide another characterization.

#### Proposition 11

Let (*G*, *y*) be a reaction network decomposed into $\ell$ subnetworks. Then $d = d_1 + \cdots + d_{\ell }$ if and only if $L=L_1 \oplus \cdots \oplus L_{\ell }$.

#### Proof

Recall $d = |V^\sqcup _s| - \ell - \dim (L)$ and $d_j = |V^j_s| - 1 - \dim (L_j)$. Clearly, $V_s^\sqcup = V_s^1 \sqcup \cdots \sqcup V_s^\ell$ implies $|V_s^\sqcup | = |V_s^1| + \cdots + |V_s^\ell |$. Hence,

$$ d = |V^\sqcup _s| - \ell - \dim (L) = \sum _{j=1}^\ell d_j = \sum _{j=1}^\ell \left( |V^j_s| - 1 - \dim (L_j) \right) $$

if and only if $\dim (L) =\sum _{j=1}^\ell \dim (L_j)$, that is, $L = L_1 \oplus \cdots \oplus L_\ell$. $\square$

For a summary of the notation introduced in this section, see Table 1(bc). For an illustration, we continue the example from Sects. 2 and 4.

#### Example

*(continued)* For the last time, we return to the reaction network (with rate constants)The decomposition of the kernel of the stoichiometric matrix $$N = \begin{pmatrix} N^1&N^2 \end{pmatrix}$$ withas a direct product, that is, $\ker N = \ker N^1 \times \ker N^2$ implies a partition of the edge set, $E = E^1 \dot{\cup }E^2$ with $E^1 = \{12,21,43\}$ and $E^2 = \{45,56,65\}$, and hence a decomposition of the reaction network (*G*, *y*) into $\ell =2$ independent subnetworks. 
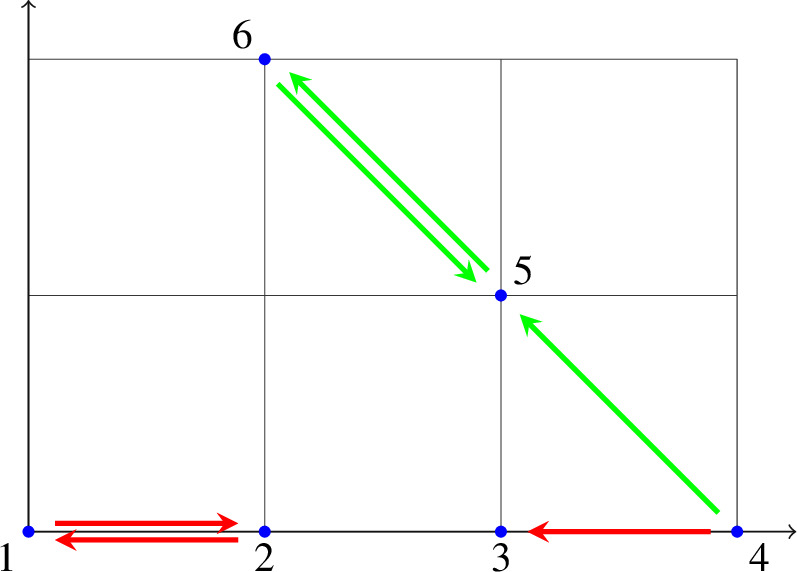
 In the “embedded” graph, the subnetworks $(G^1,y^1)$ and $(G^2,y^2)$ are shown in red and green, respectively. Note that $G^1=(V^1,E^1)$ has vertex set $V^1 = \{ 1,2,3,4 \}$, whereas $V^2 = \{ 4,5,6 \}$ for $G^2=(V^2,E^2)$, and hence $V^1 \cap V^2 = \{4\} \ne \emptyset$. Accordingly, we introduce the disjoint union of vertex sets

$$\begin{aligned} V^\sqcup = V^1 \sqcup V^2 = \{ 1,2,3,4^1,4^2,5,6 \}, \end{aligned}$$

which contains two “copies” of the element in the intersection, $4 \in V^1 \cap V^2$, and the disjoint union of the source vertex sets $V_s^\sqcup = V_s^1 \sqcup V_s^2 = \{ 1,2,4^1,4^2,5,6 \}$.

From the matrices for the subnetworks, we define the corresponding block(-diagonal) combined matrices: the combined $V^\sqcup \times E$ incidence and $V_s^\sqcup \times E$ source matrices,the combined $V^\sqcup \times V_s^\sqcup$ (rectangular) Laplacian matrix,the combined $n \times V^\sqcup$ complex and $n \times V_s^\sqcup$ source complex matrices,and the combined $n \times V_s^\sqcup$ kinetic matrix,The latter defines the combined kinetic subspace, $K^* = {{\,\textrm{im}\,}}\Gamma _k^* = \mathbb {R}^2$ (for all *k*).

**Table 1:** Notation introduced in Sects. 4 and 5. (a) Matrices and subspaces for a reaction network (*G*, *y*) / a mass-action system $(G_k,y)$ with underlying graph $G=(V,E)$ and edge labels *k*. (b) Network decomposition: objects in (a) for subnetworks $(G^j,y^j)$ with $G^j=(V^j,E^j)$ and combined objects for the full network (*G*, *y*). (c) Polytope and subspaces for the parametrized system of polynomial equations $\Gamma _k^* \, x^{Y^*_s} = 0$, arising from the decomposed dynamical system (4). See Definition 1 for $A \left( c\circ x^B \right) = 0$ with $A=\Gamma _k^*$, $B=Y^*_s$, and $c=1$; analogously for the subsystems $\Gamma _k^j \, x^{Y_s^j} = 0$

symbol	dimension	name	relation
*(a) reaction network*			
$I_E$	$V \times E$	incidence matrix	
$I_{E,s}$	$V_s \times E$	source matrix	
$R_k$	$V \times V_s$	(rectangular) Laplacian matrix	$R_k = I_E \text {diag}(k) (I_{E,s})^T$
*Y*	$n \times V$	complex matrix	
$Y_s$	$n \times V_s$	source complex matrix	
*N*	$n \times E$	stoichiometric matrix	$N = Y I_E$
$\Gamma _k$	$n \times V_s$	kinetic matrix	$\Gamma _k = Y R_k$
*S*		stoichiometric subspace	$S = \text {im } N$
*K*		kinetic subspace	$K = \text {im } \Gamma _k$
*l*		number of connected components	
*t*		number of absorbing strong comp	
$\delta$		deficiency	$\delta = |V| - l - \dim (S)$
*(b) network decomposition*			
$\ell$		number of (independent) subnetworks	
$I_E^j, I_{E,s}^j, R_k^j, Y^j, \dots$		objects in (a) for $j = 1, \dots , \ell$	
$V^\sqcup$		disjoint union of vertex sets	$V^\sqcup = V^1 \sqcup \dots \sqcup V^\ell$
$V_s^\sqcup$		disjoint union of source vertex sets	$V_s^\sqcup = V_s^1 \sqcup \dots \sqcup V_s^2$
$I_E^*$	$V^\sqcup \times E$	combined incidence matrix	
$I_{E,s}^*$	$V_s^\sqcup \times E$	combined source matrix	
$R_k^*$	$V^\sqcup \times V_s^\sqcup$	combined (rect.) Laplacian matrix	$R_k^* = I_E^* \text {diag}(k) (I_{E,s}^*)^T$
$Y^*$	$n \times V^\sqcup$	combined complex matrix	
$Y_s^*$	$n \times V_s^\sqcup$	combined source complex matrix	
$\Gamma _k^*$	$n \times V_s^\sqcup$	combined kinetic matrix	$\Gamma _k^* = Y^* R_k^*$
$K^*$		combined kinetic subspace	$K^* = \text {im } \Gamma _k^*$
*(c) polynomial equations*			
*P*		coefficient polytope	
*L*		monomial difference subspace	
*D*		monomial dependency subspace	
*d*		monomial dependency	$d = \dim D$
$P_j, L_j, D_j, d_j$		objects for $j = 1, \dots , \ell$	

## Dependency One Mass-Action Systems

We present a *dependency* one theorem for mass-action systems, extending the deficiency one theorems by Feinberg (Feinberg 1987, 1995a), cf. Theorem 18.

In Theorem 14 below, we neither assume $\delta _i \le 1$, $t_i = 1$, nor $K=S$. Most importantly, we need to guarantee $d = \dim P$ (per class), in order to apply Theorem 8.

### Lemma 12

Let (*G*, *y*) be a reaction network with one subnetwork (the network itself) and let $(G_k,y)$ be the corresponding mass-action system. Consider the equilibrium equation $\Gamma _k \, x^{Y_s} = 0$ (with one class) and assume $\ker \Gamma _k \cap \mathbb {R}^{V_s}_> \ne \emptyset$. Then,

$$ \dim P = d \quad \text {if and only if} \quad \dim K = \dim L. $$

### Proof

On the one hand, $\dim P + 1 = \dim C = \dim (\ker \Gamma _k) = \dim (\ker (Y R_k)) = |V_s| - \dim ({{\,\textrm{im}\,}}(Y R_k)) = |V_s| - \dim K$. On the other hand, $d + 1 = |V_s| - \dim L$. Hence, $\dim P = d$ if and only if $\dim K = \dim L$. $\square$

As stated above, we do not explicitly restrict the number of absorbing strong components (per class), in particular, if they are singletons. However, we record that $\dim P \le 1$ (an at most one-dimensional coefficient polytope) implies $t' \le 2$ (at most two non-singleton absorbing strong components).

### Fact 13

Let (*G*, *y*) be a reaction network with one subnetwork (the network itself) and let $(G_k,y)$ be the corresponding mass-action system. Consider the equilibrium equation $\Gamma _k \, x^{Y_s} = 0$ (with one class) and assume $\ker \Gamma _k \cap \mathbb {R}^{V_s}_> \ne \emptyset$. Then,

$$ \dim P \le 1 \implies {t' \le 2.} $$

In fact, $t'=2$ only if there are two components ($l=2$) which are strongly connected.

### Proof

Let $\dim P \le 1$. Then, $t' \le 2$ since

$$\begin{aligned} \dim P+1&= \dim C = \dim (\ker \Gamma _k) = \dim (\ker (Y R_k)) \\&= \dim (\ker Y \cap {{\,\textrm{im}\,}}R_k) + \underbrace{\dim (\ker R_k)}_{t'} . \end{aligned}$$

If $t' = 2$, then $\ker \Gamma _k = \ker R_k$ is generated by two non-negative vectors with support on the two non-singleton absorbing strong components. Since the union of their supports is the set of all source vertices, there are two components, and they are strongly connected. $\square$

### Theorem 14

($d_j\le 1$, mass-action systems) Let $(G_k,y)$ be a mass-action system with $\ell$ independent subnetworks. Recall the kinetic and stoichiometric subspaces, *K* and *S*, respectively. For the equilibrium equation $\Gamma ^*_k \, x^{Y_s^*} = 0$, recall the monomial dependency *d* and the monomial difference subspace *L*. Let $(G_k,y)$ fulfill the following conditions:

- $$\ker \Gamma _k^* \cap \mathbb {R}^{V^\sqcup _s}_> \ne \emptyset $$.
- $$d = d_1 + \cdots + d_{\ell }$$.
- For every (class) $j=1,\ldots ,\ell$,
  $$d_j \le 1$$ and $$\dim (K_j)= \dim (L_j)$$.
  If $$d_j=1$$, then
    $\displaystyle \sum _{i'=1}^{i} \bar{b}^j_{i'} \ge 0$ for all $i=1,2,\ldots ,\omega _j-1$ (or “$\le 0$” for all *i*)
    $$\bar{b}^j_1 \cdot \bar{b}^j_{\omega _j} < 0$$.
    (The components of the (lumped) dependency vector  are ordered with respect to the vector $\bar{q}^j \in \mathbb {R}^{\omega _j}$ of the polytope $P_j$, see Definition 7.)
- $$L=K$$ or (IVb) $$L=S$$.

Then, there exists a unique positive equilibrium within every (IVa) kinetic or (IVb) stoichiometric compatibility class.

### Proof

The mass-action system $(G_k,y)$ gives rise to the polynomial equations $\Gamma _k^* \, x^{Y_s^*} = 0$ with $\ell$ classes. In order to apply Theorem 8 (for $A \left( c \circ x^B \right) = 0$ with $A=\Gamma _k^*$, $B=Y^*_s$, and $c=1$), we show that conditions (i), (ii), (iii) there follow from conditions (I), (II), (III) here.

(i) By (I) with $$A = \Gamma _k^*$$.

(ii) By (II).

(iii) By (III), $\dim (K_j)= \dim (L_j)$, and by Lemma 12, $d_j = \dim P_j$ (for $j=1,\ldots ,\ell$). Regarding $d_j=1$, (iii) and (III) are identical.

By Theorem 8, the solution set on the coefficient polytope is a singleton, $|Y_c|=1$, and by Theorem 2, the solution set is an exponential fiber, $Z_c = x^* \circ {{\,\textrm{e}\,}}^{L^\perp }$ with $x^* \in \mathbb {R}^n_>$.

The set of positive equilibria within the kinetic compatibility class given by $x' \in \mathbb {R}^n_>$ is $x^* \circ {{\,\textrm{e}\,}}^{L^\perp } \cap \, (x' + K)$. By (IVa), $K=L$, and by Theorem 3, the intersection is a singleton.

The set of positive equilibria within the stoichiometric compatibility class given by $x' \in \mathbb {R}^n_>$ is $x^* \circ {{\,\textrm{e}\,}}^{L^\perp } \cap \,(x' + S)$. By (IVb), $L=S$, and by Theorem 3, the intersection is a singleton. $\square$

We now provide a simplified version of Theorem 14 that is closer to the (extended) deficiency one theorem (Theorem 18 in Sect. 7.1). In particular, we assume that the kinetic, stoichiometric, and monomial difference subspaces of the subnetworks (and hence of the full system) agree.

### Corollary 15

Let $(G_k,y)$ be a mass-action system with $\ell$ independent subnetworks. Recall the kinetic and stoichiometric subspaces, *K* and *S*, respectively. For the equilibrium equation $\Gamma ^*_k \, x^{Y_s^*} = 0$, recall the monomial dependency *d* and the monomial difference subspace *L*. Let $(G_k,y)$ fulfill the following conditions:

- (I’) $$\ker \Gamma _k^* \cap \mathbb {R}^{V^\sqcup _s}_> \ne \emptyset $$.
- (III’) For every (independent subnetwork) $j=1,\ldots ,\ell$,
  - $$d_j \le 1$$ and $$K_j = S_j = L_j$$.
  - If $$d_j=1$$, then
    - $\displaystyle \sum _{i'=1}^{i} \bar{b}^j_{i'} \ge 0$ for all $i=1,2,\ldots ,\omega _j-1$ (or “$\le 0$” for all *i*)
    - $$\bar{b}^j_1 \cdot \bar{b}^j_{\omega _j} < 0$$.
    (The components of the (lumped) dependency vector $\bar{b}^j \in \mathbb {R}^{\omega _j}$ are ordered with respect to the vector $\bar{q}^j \in \mathbb {R}^{\omega _j}$ of the polytope $P_j$, see Definition 7.)

Then, there exists a unique positive equilibrium within every stoichiometric compatibility class.

### Proof

The mass-action system $(G_k,y)$ gives rise to the polynomial equations $\Gamma _k^* \, x^{Y_s^*} = 0$ with $\ell$ classes (determined by the $\ell$ independent subnetworks), cf. Proposition 10. In order to apply Theorem 14, we show that conditions (I), (II), (III), (IVb) there follow from conditions (I’), (III’) here.

(I) By (I’).

(II) By (III’), $L_j=S_j$, and by the decomposition of $\ker N$, $S=S_1 \oplus \cdots \oplus S_{\ell }$. Hence, $L=L_1 \oplus \cdots \oplus L_{\ell }$, and (II) by Proposition 11.

(III) By (III’).

(VIb) By (III’), see the argument for (II). $\square$

## Deficiency One Mass-Action Systems

In the setting of the deficiency one theorem, we assume that the network is decomposed into $\ell$ independent subnetworks with one (absorbing) strong component. That is, the classes arise from $\ker N$ and they are assumed to be *connected*. This implies $\delta = \delta _1 + \cdots + \delta _\ell$, see Fact 17. The main result of this subsection is Lemma 16, a graph-theoretical argument used in the proof for the existence of a positive equilibrium in the case of weakly reversible networks, see Theorem 18 in Subsection 7.1 and its proof in Subsection 7.3.

Given the directed graph $G=(V,E)$ of a network, we introduce the bipartite graph $\mathcal {G} = ({\mathcal {C}},{\mathcal {V}},\mathcal {E})$ with the first vertex set ${\mathcal {C}}= \{1,\ldots ,\ell \}$ representing subnetworks of *G*, the second vertex set ${\mathcal {V}}\subseteq V$ representing “shared” vertices (appearing in more than one subnetwork), and the edge set $\mathcal {E} = \{ (j,i) \in {\mathcal {C}}\times {\mathcal {V}}\mid i \in V^j \}$, connecting shared vertices with their subnetworks.

### Lemma 16

Let (*G*, *y*) with $G=(V,E)$ be a reaction network decomposed into $\ell$ connected, independent subnetworks. Recall the (combined) incidence matrices, $I_E$ and $I_E^*$, the number *l* of connected components, and the disjoint union $V^\sqcup$ of source vertex sets. Then, $|V^\sqcup | - |V| = \ell - l$, that is, $\ker I_E = \ker I_E^*$.

### Proof

Since we assume that subgraphs are connected, all vertices of a given subgraph derive from only one component of the original graph, and we can consider the decomposition of $l=1$ component into $\ell$ subgraphs. For the resulting bipartite graph $\mathcal {G}$, $|{\mathcal {C}}| = \ell$ and

$$ |\mathcal {E}| = \sum _{i \in {\mathcal {V}}} \text {deg}(i) = \sum _{i \in {\mathcal {V}}} (\text {deg}(i) - 1 + 1) = V^\sqcup - V + |{\mathcal {V}}|. $$

Since $\mathcal {G}$ is connected, $|\mathcal {E}| \ge |{\mathcal {C}}| + |{\mathcal {V}}| -1$, that is, $V^\sqcup - V \ge \ell - 1$. For clarity, note that a “double edge” $(i j i')$ indicates that the shared vertices $i, i'$ reside in subgraph *j* and hence $0 \ne y(i')-y(i) \in S_j$. Now, assume $V^\sqcup - V > \ell - 1$. Then, there is a cycle of $n\ge 2$ “double edges” with vertex sequence $i_1 j_1 i_2 j_2 \ldots i_n j_n i_{n+1}$, where $i_{n+1}=i_1$, $i_* \in {\mathcal {V}}\subseteq V$, $j_* \in {\mathcal {C}}= \{1,\ldots ,\ell \}$. Hence,

$$ \underbrace{y(i_2) - y(i_1)}_{\in S_{j_1}} + \cdots + \underbrace{y(i_1) - y(i_n)}_{\in S_{j_n}} = 0, $$

contradicting $S = S_1 \oplus \cdots \oplus S_\ell$. Hence, $V^\sqcup - V = \ell - 1$. By combining the arguments for the *l* components of the original graph, $V^\sqcup - V = \ell - l$.

Recall $I_E \in \{-1,0,1\}^{V \times E}$ and $I_E^* \in \{-1,0,1\}^{V^\sqcup \times E}$. Obviously, $\ker I_E \supseteq \ker I_E^*$. By the rank-nullity theorem, $\dim (\ker I_E) = |E| - \dim ({{\,\textrm{im}\,}}I_E) = |E| - \dim ({{\,\textrm{im}\,}}I_E^\textsf{T}) = |E| - |V| + \dim (\ker I_E^\textsf{T}) = |E| - |V| + l$ as well as $\dim (\ker I_E^*) = |E| - |V^\sqcup | + \ell$. Hence, $\dim (\ker I_E) - \dim (\ker I_E^*) = |V^\sqcup | - |V| - (\ell -l)$. $\square$

### Fact 17

Let (*G*, *y*) be a reaction network decomposed into $\ell$ connected subnetworks. The following statements are equivalent.

1. $$S = S_1 \oplus \cdots \oplus S_\ell $$.
2. $$\delta = \delta _1 + \cdots + \delta _\ell $$ and $$|V^\sqcup | - |V| = \ell - l$$.

### Proof

$2 \implies 1$. On the one hand, $\delta = |V|-l-\dim S$. On the other hand, $\delta _j = |V^j| - 1 - \dim S_j$ and hence $\sum _j \delta _j = |V^\sqcup | - \ell - \sum _j \dim S_j$. Altogether,

$$ \sum _j \dim S_j - \dim S = \delta - \sum _j \delta _j + |V^\sqcup | - |V| - (\ell - l). $$

$1 \implies 2$. By Lemma 16, if $S = S_1 \oplus \cdots \oplus S_\ell$, then $|V^\sqcup | - |V| = \ell - l$. By the displayed equation above, also $\delta = \sum _j \delta _j$. $\square$

In the “classical” setting ($\ell = l)$, the connected subnetworks are the components of the graph (the linkage classes), and hence they are independent exactly when their deficiencies add up to the network deficiency.

### The (extended) deficiency one theorem

We prove Theorem 18 below which already extends the “classical” deficiency one theorem (Feinberg 1987, Theorem 6.2.1) from components (“linkage classes”) to independent subnetworks, cf. (Feinberg 1987, Remark 6.2.D) and (Feinberg 1995a, Theorem A.1).

#### Theorem 18

($\delta _j \le 1$, independent subnetworks) Let $(G_k,y)$ be a mass-action system with $\ell$ independent subnetworks. Recall the deficiency $\delta$ and the number *t* of absorbing strong components. Let $(G_k,y)$ fulfill the following conditions:

For every (independent subnetwork) $j=1,\ldots ,\ell$,

(i) $$\delta _j\le 1$$ and $$t_j=1$$.

If (ii) there exists a positive equilibrium, then there exists a unique positive equilibrium within every stoichiometric compatibility class.

If the system is weakly reversible, then (ii) for all rate constants *k*.

#### Remark

*(on the statement)* Note that in the deficiency one theorems, the existence of a positive equilibrium is assumed. In our dependency one theorem, we only assume the necessary condition $\ker \Gamma _k^* \cap \mathbb {R}^{V^\sqcup _s}_> \ne \emptyset$. Indeed, in Theorem 18, the existence assumption can be replaced by the necessary condition. A different (more involved) condition was given by (Boros 2013b).

#### Remark

*(on the proof)* A detailed proof of the classical theorem is given in (Feinberg 1995a, Sections 5–8 and Appendix B). For an outline of the proof of the extension (to independent subnetworks or “direct partitions”), see (Feinberg 1995a, Appendix A). In (Feinberg (1995a), Sections 5 and 6), Feinberg shows that the set of positive equilibria has the form $x^* \circ e^{S^\perp }$ (a particular solution $x^*$ times the exponentiation of the orthogonal complement of the stoichiometric subspace *S*), by reformulating the problem using a monotonic function. Our approach is based on a geometric framework for parametrized systems of polynomial equations and inequalities (Müller and Regensburger 2023, 2026). Within this framework, we establish the existence of a point $y^*$ on the coefficient polytope (where $y^*$ is in one-to-one correspondence with $x^* \circ e^{S^\perp }$). As Feinberg, we use the “second salt theorem” (a network argument) and a version of “Birch’s theorem”. In the case of weakly reversible networks, we replace the proof for the existence of a positive equilibrium in (Feinberg 1995a, Section 8) by a graph-theoretical argument (see the last paragraph in the proof of Theorem 18, which uses Lemma 16).

In Subsection 7.3, we will show that the deficiency one theorem follows from our more general dependency one results, Theorem 14 or its Corollary 15. Indeed, we exhibit how the network conditions in Theorem 18 ensure the conditions in Corollary 15.

First, we show that the kinetic, stoichiometric, and monomial difference subspaces of the subnetworks agree.

#### Lemma 19

Let $(G_k,y)$ be a mass-action system with $t=1$ (one absorbing strong component). Recall the number $t'$ of non-singleton absorbing strong components and the deficiency $\delta$. Further, recall the kinetic and stoichiometric subspaces, *K* and *S*, respectively. For the equilibrium equation $\Gamma _k \, x^{Y_s} = 0$, recall the dependency *d* and the monomial difference subspace *L*, and assume that $\ker \Gamma _k \cap \mathbb {R}^{V_s}_> \ne \emptyset$. Then,

1. $$K=L=S$$.
2. $$d = \delta + t '-1$$.

#### Proof

1. Since $t=l=1$, ${{\,\textrm{im}\,}}R_k = {{\,\textrm{im}\,}}I_E$ and hence $K = {{\,\textrm{im}\,}}(Y R_{k}) = {{\,\textrm{im}\,}}(Y I_{E}) = S$.

If $t'=1$ (the absorbing strong component is not a singleton), then $V_s = V$ and $L=S$. If $t'=0$ (the absorbing strong component is a singleton), then $V = V_s \dot{\cup }\{i_*\}$ and $E = E_s \dot{\cup }E_*$, where $E_s$ is the set of reactions between source vertices (and hence $E_*$ is the set of reactions with target vertex $i_*$).

Now, note that *S* is the linear subspace associated with the affine hull of the complexes, ${{\,\textrm{aff}\,}}(Y) = {{\,\textrm{aff}\,}}(y(i) \mid i \in V)$, and *L* is the linear subspace associated with the affine hull of the source complexes, ${{\,\textrm{aff}\,}}(Y_s) = {{\,\textrm{aff}\,}}(y(i) \mid i \in V_s)$. We will show $y(i_*) \in {{\,\textrm{aff}\,}}(Y_s)$ which implies ${{\,\textrm{aff}\,}}(Y_s) = {{\,\textrm{aff}\,}}(Y)$ and hence $L=S$.

Since $\ker \Gamma _k \cap \mathbb {R}^{V_s}_> \ne \emptyset$, there is $\xi \in \mathbb {R}^{V_s}_>$ such that $\Gamma _k \, \xi = 0$, and hence there is $\alpha = {{\,\textrm{diag}\,}}(k) (I_{E,s})^\textsf{T}\xi \in \mathbb {R}^E_>$ such that $Y I_E \, \alpha = 0$. That is, $Y I_{E_s} \alpha _{E_s} + Y I_{E_*} \alpha _{E_*} = 0$, using $$I_E = \begin{pmatrix} I_{E_s}&I_{E_*} \end{pmatrix}$$ and $$\alpha = \left( {\begin{array}{c}\alpha _{E_s}\\ \alpha _{E_*}\end{array}}\right) $$. Clearly, $Y I_{E_*} \alpha _{E_*} = - Y I_{E_s} \alpha _{E_s} \in L$. Explicitly,

$$ \delta y:= Y I_{E_*} \alpha _{E_*} \!=\! \sum _{(i \rightarrow i_*) \in E_*} \left( y(i_*) - y(i) \right) \alpha _{i \rightarrow i_*} \!=\! \bar{\alpha } \Bigg ( y(i_*) - \underbrace{\sum _{(i \rightarrow i_*) \in E_*} \beta _{i \rightarrow i_*} \, y(i)}_{y} \Bigg ), $$

where $\bar{\alpha } = \sum _{(i \rightarrow i_*) \in E_*} \alpha _{i \rightarrow i_*}$, $\beta _{i \rightarrow i_*} = \alpha _{i \rightarrow i_*} / \bar{\alpha }$, and $\sum _{(i \rightarrow i_*) \in E_*} \beta _{i \rightarrow i_*} = 1$. That is, $y(i_*) = y + \delta y / \bar{\alpha }$, where $y \in {{\,\textrm{aff}\,}}(Y_s)$ and $\delta y \in L$. Hence, also $y(i_*) \in {{\,\textrm{aff}\,}}(Y_s)$.

2. Recall $d = |V_s| - 1 - \dim L$ and $\delta = |V| - 1 - \dim S$. By statement 1, $L=S$ and hence $d -\delta = |V_s| - |V| = t -t' = 1 - t'$. Hence $d = \delta + t'-1$. $\square$

Regarding dimensions, the assumption $t=1$ has two consequences: if $t'=1$ (non-singleton absorbing strong component), then $d=\delta$; however, if $t'=0$ (singleton absorbing strong component), then $d=\delta -1$. In particular, $\delta =1$ and $t'=0$ imply $d=0$.

Second, to show that the sign conditions (for the dependency vector) in Corollary 15 are fulfilled, we provide a variant of Feinberg’s “second salt theorem” (Feinberg 2019).

### The Second Salt Theorem

We continue our study of subnetworks (with $\delta \le 1$, $t=1$, and $\ker (Y R_k) \cap \mathbb {R}^{V_s}_> \ne \emptyset$). By Lemma 12 and Lemma 19, $d=\dim P\le 1$. We consider the nontrivial case $d=\dim P=1$ which has $\delta =t'=1$, again by Lemma 19.

Let $y^1, y^2 \in \ker (Y R_k) \cap \mathbb {R}^{V_s}_\ge$ be the vertices of *P*. Since $t'=1$, there is a non-negative $\hat{y} \in {\overline{P}}$ with $R_k \, \hat{y} = 0$ and support on the (non-singleton) absorbing strong component. (It lies in the interior of the coefficient polytope, if the network is weakly reversible, and on the boundary, otherwise.) Since $\delta = 1$, there is a positive $\bar{y} \in P$ with $R_k \, \bar{y} = \beta \in \mathbb {R}^V$ and ${{\,\textrm{im}\,}}\beta = \ker Y \cap {{\,\textrm{im}\,}}R_k$. (It lies in the interior, possibly after adding $\hat{y}$.) That is,

7 $$\begin{aligned} \begin{aligned} R_k \, \hat{y}&= 0 , \\ R_k \, \bar{y}&= \beta . \end{aligned} \end{aligned}$$

Without loss of generality, we assume that the vertices $y^1$, $y^2$ of the coefficient polytope (a line segment) and the vectors $\hat{y}$, $\bar{y}$ are ordered as $y^1 \cdots \hat{y} \cdots \bar{y} \cdots y^2$, where $\hat{y}=y^1$ if the network is not weakly reversible. Now, $\hat{y} = \hat{\lambda } y^1 + (1- \hat{\lambda }) y^2$ for some $\hat{\lambda } \in (0,1]$ and $\bar{y} = \bar{\lambda } y^1 + (1- \bar{\lambda }) y^2$ for some $\bar{\lambda } \in (0,1)$, where $\hat{\lambda } > \bar{\lambda }$. Let

$$ \hat{q} = \hat{y} \circ (\bar{y})^{-1} = \left( \hat{\lambda } y^1 + (1- \hat{\lambda }) y^2 \right) \circ \left( \bar{\lambda } y^1 + (1- \bar{\lambda }) y^2 \right) ^{-1}. $$

It is easy to see^2^ that the order of the entries of ${\hat{q}}$ agrees with the order of the entries of

$$ q = \left( y^1-y^2\right) \circ \left( y^1+y^2\right) ^{-1} $$

specified in Definition 4.

Finally, we introduce the scaled rectangular graph Laplacian

$$ R_k {{\,\textrm{diag}\,}}(\bar{y}) = R_{\bar{k}} \quad \text {with} \quad \bar{k} = k \circ \bar{y}, $$

and we rewrite Equations (7) as

8 $$\begin{aligned} \begin{aligned} R_{\bar{k}} \, \hat{q}&= 0 , \\ R_{\bar{k}} \, 1_{V_s}&= \beta . \end{aligned} \end{aligned}$$

Now, we state Feinberg’s “second salt theorem” (Feinberg 2019) for the rectangular (rather than the square) graph Laplacian.

#### Lemma 20

Let $G_k=(V,E,k)$ be a labeled simple digraph with one component. Let $T = \{1,\ldots ,t\} \subseteq V = \{1,\ldots ,m\}$ be (the vertices of) an absorbing strong component, let $\hat{q} \in \mathbb {R}^{V_s}_\ge$ be the corresponding vector in the kernel of the rectangular graph Laplacian, that is, $R_k \, \hat{q} = 0$ and ${{\,\textrm{supp}\,}}\hat{q} = T$, and assume $\hat{q}_1 \ge \cdots \ge \hat{q}_t > \hat{q}_{t+1} = \cdots = \hat{q}_m = 0$. Further, let $\beta = R_k \, 1_{V_s} \in \mathbb {R}^V$. Then,

$$ \sum _{i'=1}^i \beta _{i'} \ge 0, \quad \text {for } i \in T. $$

If $i < t$ and $\hat{q}_{i} > \hat{q}_{i+1}$, then $\sum _{i'=1}^i \beta _{i'} > 0$. Finally, $\sum _{i'=1}^t \beta _{i'} = 0$ if and only if $T=V$.

#### Proof

See Appendix B.2. $$\square $$

### Proof of the extended deficiency one theorem (for independent subnetworks)

#### Proof of Theorem 18

The mass-action system $(G_k,y)$ gives rise to the polynomial equations $\Gamma _k^* \, x^{Y_s^*} = 0$ with $\ell$ classes (determined by the $\ell$ connected, independent subnetworks), cf. Proposition 10. In order to apply Corollary 15, we show that conditions (I’), (III’) there follow from conditions (i), (ii) here.

(I’) If (ii), there exists a positive equilibrium, $x \in \mathbb {R}^n_>$ with $\Gamma _k^* \, x^{Y_s^*} = 0$, and hence (I’). This further implies $\ker \Gamma _k^j \cap \mathbb {R}^{V^j_s}_> \ne \emptyset$ for the mass-action systems $(G^j_k,y^k)$, $j=1,\ldots ,\ell$, having $t_j=1$ by (i). Hence, Lemma 19 applies to the subsystems.
(III’) By Lemma 19, $K_j = S_j = L_j$ and $d_j \le 1$, $j=1,\ldots ,\ell$. It remains to consider $d_j=1$ in detail, implying $\delta _j=1$ and $t'_j=1$; in particular, all vertices are source vertices, that is, $V^j_s = V^j$. By the argument before Lemma 20, there are $\beta \in \mathbb {R}^{V^j}$ with ${{\,\textrm{im}\,}}\beta = \ker Y^j \cap {{\,\textrm{im}\,}}R^j_k$ and $\hat{q} \in \mathbb {R}^{V^j_s}_\ge$ such that $R^j_{\bar{k}} \, \hat{q}=0$ and $R^j_{\bar{k}} \, 1=\beta$ (for a certain $\bar{k} \in \mathbb {R}^{V^j_s}_>$), cf. Equation (8). In particular, ${{\,\textrm{supp}\,}}\hat{q} = T$, where $T \subseteq V^j$ denotes (the vertices of) the absorbing strong component. Let $t=|T|$ and $m=|V^j|$ and assume $\hat{q}_1 \ge \cdots \ge \hat{q}_t > \hat{q}_{t+1} = \cdots = \hat{q}_m = 0$. By Lemma 20, $\sum _{i'=1}^i \beta _{i'} \ge 0, \text { for } i = 1,\ldots ,t.$ Further, if $i < t$ and $\hat{q}_{i} > \hat{q}_{i+1}$, then $\sum _{i'=1}^i \beta _{i'} > 0$. Finally, $\sum _{i'=1}^t \beta _{i'} = 0$ if and only if $T=V^j$. The polynomial system $\Gamma _k^j \, x^{Y^j_s}=0$ is of the form ${A \, (c \circ x^B)=0}$ with $B = Y^j_s = Y^j$, and the dependency vector $b \in \mathbb {R}^{V^j}$ in Corollary 15 is given by $${{\,\textrm{im}\,}}b = D_k = \ker \left( {\begin{array}{c}B\\ 1^\textsf{T}\end{array}}\right) = \ker \left( {\begin{array}{c}Y^j\\ 1^\textsf{T}\end{array}}\right) $$. Since ${{\,\textrm{im}\,}}R^j_k = {{\,\textrm{im}\,}}I_E^j = \ker 1^\textsf{T}$, equivalently ${{\,\textrm{im}\,}}b = \ker Y^j \cap {{\,\textrm{im}\,}}R^j_k$. That is, we can choose $b=\beta$ and the (in)equalities for $\beta$ guaranteed by Lemma 20 also hold for *b*. Now, recall that $I_1, \ldots , I_\omega \subset V^j$ denote $\omega$ equivalence classes corresponding to equal (consecutive) components of $\hat{q}$ and that $\bar{b} \in \mathbb {R}^\omega$ with $\bar{b}_i = \sum _{i' \in I_i} b_{i'}$ is the vector of lumped *b*’s. Since $\sum _{i'=1}^i b_{i'} \ge 0$ for $i=1,\ldots ,t$, we get ${\sum _{i'=1}^i \bar{b}_{i'} {>} 0}$ for $i=1,2,\ldots ,\omega -1$. (If $T \subset V^j$ and hence $I_\omega = \{t+1,\ldots ,m\}$, then $\sum _{i'=1}^{\omega -1} \bar{b}_{i'} = \sum _{i'=1}^t b_{i'} > 0$.) Finally, since $\sum _{i=1}^\omega \bar{b}_i = 0$, we get $\bar{b}_\omega < 0$ and hence $\bar{b}_1\cdot \bar{b}_\omega <0$.

By Corollary 15, there exists a unique positive equilibrium within every stoichiometric compatibility class.

It remains to show that weak reversibility implies (ii). By Lemma 16, $\ker I_E = \ker I_E^*$, and hence the cycles of the graph *G* agree with the cycles of the subgraphs $G^j$, $j=1,\ldots ,\ell$. Hence, if *G* is weakly reversible, then every $G^j$ is weakly reversible and $\ker R^j_k \cap \mathbb {R}^{V_s}_> \ne \emptyset$. Since $\Gamma _k^j = Y^j R^j_k$, also $\ker \Gamma _k^j \cap \mathbb {R}^{V_s}_> \ne \emptyset$, and since $$\Gamma _k^* = \begin{pmatrix} \Gamma ^1_k&\ldots&\Gamma ^\ell _k \end{pmatrix}$$, also $\ker \Gamma _k^* \cap \mathbb {R}^{V^\sqcup _s}_> \ne \emptyset$. That is, (ii). $\square$

## Examples

### Example 1

**Fig. 1:**
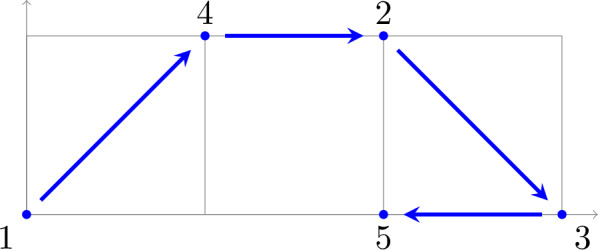
Reaction network (as an embedded graph) with 5 vertices and 4 source vertices and hence $\delta = 5-1-2 = 2$, but $d = 4-1-2 = 1$

**Deficiency two** network with one (singleton) absorbing strong component as shown in Fig. 1.

The ODE associated with the mass-action system is given by

$$ \begin{aligned} \frac{\text {d} x_1}{\text {d} t}&= k_{14} + k_{23} \, x_1^2 x_2 - k_{35} \, x_1^3 + k_{42} \, x_1 x_2 , \\ \frac{\text {d} x_2}{\text {d} t}&= k_{14} - k_{23} \, x_1^2 x_2 , \end{aligned} $$

and steady state can be written as $\Gamma _k \, x^{Y_s}=0$ with $$\Gamma _k = \begin{pmatrix} k_{14} & k_{23} & -k_{35} & k_{42} \\ k_{14} & -k_{23} & 0 & 0 \end{pmatrix}$$ and $$Y_s = \begin{pmatrix} 0 & 2 & 3 & 1 \\ 0 & 1 & 0 & 1 \end{pmatrix}$$. We show that conditions (I), (II), (III), and (IVb) of Theorem 14 are satisfied.

(I) $\ker \Gamma _k\cap \mathbb {R}^4_{>0}$ is generated by $y^1 = ( \frac{1}{k_{14}}, \frac{1}{k_{23}}, \frac{2}{k_{35}}, 0 )^\textsf{T}$ and $y^2 = ( 0, 0, \frac{1}{k_{35}}, \frac{1}{k_{42}} )^\textsf{T}$ and hence $\ker \Gamma _k\cap \mathbb {R}^4_{>0}\ne \emptyset$. As a consequence, $q = ( 1, 1, q_3, -1 )^\textsf{T}$ with $q_3 \in (-1,1)$. (The vertices of the graph *G* have been labeled such that the components of *q* are ordered.)

(II) Satisfied trivially.

(III) Clearly, $d = 4-1-2 = 1$. Since $\dim (K) = \dim ({{\,\textrm{im}\,}}{\Gamma _k})=2$, we get $K=L$. Finally, the (lumped) dependency vectors are given by $b=( 1,3,-1,-3 )^\textsf{T}$ and $\bar{b} = ( 4,-1,-3 )^\textsf{T}$ and hence $\displaystyle \sum _{i'=1}^{i} \bar{b}_{i'} \ge 0$ for $i=1,2$ as well as $\bar{b}_1\cdot \bar{b}_3 = {4\cdot (-3) < 0}$.

(IVb) Since $L = \mathbb {R}^2$ holds for the monomial difference subspace (and $L \subseteq S$), we get $L=S$.

Using Theorem 14, we find that there exists a unique positive equilibrium in every stoichiometric compatibility class, for all rate constants.

### Example 2

**Fig. 2:**
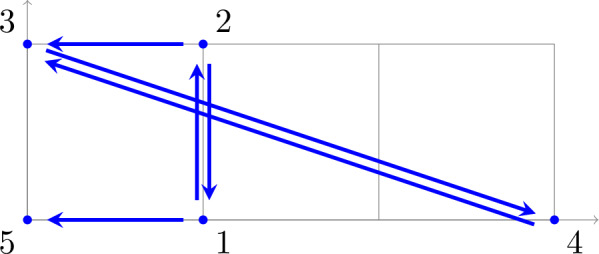
Reaction network (as an embedded graph) with 5 vertices and 4 source vertices and hence $\delta = 5-1-2 = 2$, but $d = 4-1-2 = 1$

**Deficiency two** network with **two absorbing strong components** (one singleton and one non-singleton) as shown in Fig. 2.

The ODE associated with the mass-action system is given by 
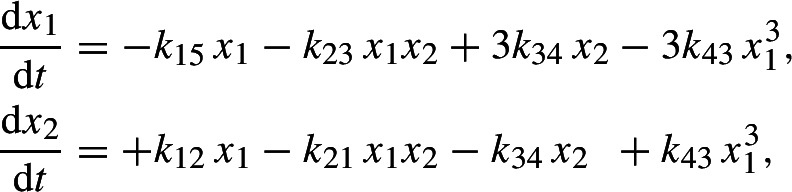
 and steady state can be written as $\Gamma _k \, x^{Y_s}=0$ with $$\Gamma _k = \begin{pmatrix} -k_{15} & -k_{23} & 3k_{34} & -3k_{43} \\ k_{12} & - k_{21} & -k_{34} & k_{43} \end{pmatrix}$$ and $$Y_s = \begin{pmatrix} 1 & 1 & 0 & 3 \\ 0 & 1 & 1 & 0 \end{pmatrix}$$. We show that conditions (I), (II), (III), and (IVb) of Theorem 14 are satisfied.

(I) $\ker \Gamma _k$ is generated by $y^1 = ( 3k_{21} + k_{23}, 3k_{12} - k_{15}, \frac{k_{12}k_{23} + k_{15}k_{21}}{k_{34}}, 0 )^\textsf{T}$ and $y^2 = ( 0, 0, \frac{1}{k_{34}}, \frac{1}{k_{43}} )^\textsf{T}$ and hence $\ker \Gamma _k\cap \mathbb {R}^4_{>0}\ne \emptyset$ iff $3k_{12} - k_{15}>0$. In this case, $q = ( 1, 1, q_3, -1 )^\textsf{T}$ with $q_3 \in (-1,1)$. (The vertices of the graph *G* have been labeled such that the components of *q* are ordered.)

(II) Satisfied trivially.

(III) Clearly, $d = 4-1-2 = 1$. Since $\dim (K) = \dim ({{\,\textrm{im}\,}}{\Gamma _k})=2$, we get $K=L$. Finally, the (lumped) dependency vectors are given by $b=(1,2,-2,-1)^\textsf{T}$ and $\bar{b} = ( 3,-2,-1 )^\textsf{T}$ and hence $\displaystyle \sum _{i'=1}^{i} \bar{b}_{i'} \ge 0$ for $i=1,2$ as well as $\bar{b}_1\cdot \bar{b}_3 = {3\cdot (-1) < 0}$.

(IVb) Since $L = \mathbb {R}^2$ holds for the monomial difference subspace (and $L \subseteq S$), we get $L=S$.

Using Theorem 14, we find that there exists a unique positive equilibrium in every stoichiometric compatibility class iff $3k_{12} - k_{15}>0$.

### Example 3

**Fig. 3:**
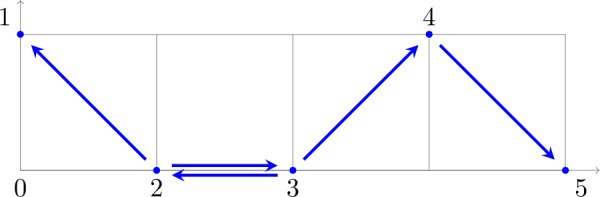
Reaction network (as an embedded graph) with 5 vertices and 3 source vertices and hence $\delta = 5-1-2 = 2$, but $d = 3-1-2 = 0$

**Deficiency two** network with **two** (singleton) **absorbing strong** components as shown in Fig. 3.

The ODE associated with the mass-action system is given by 
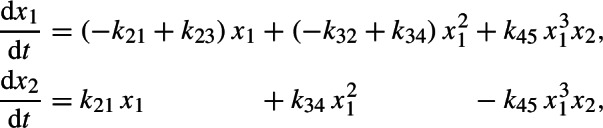
 and steady state can be written as $\Gamma _k \, x^{Y_s}=0$ with $$\Gamma _k = \begin{pmatrix} -k_{21} + k_{23} & -k_{32} + k_{34} & k_{45} \\ k_{21} & k_{34} & -k_{45} \end{pmatrix}$$ and $$Y_s = \begin{pmatrix} 1 & 2 & 3 \\ 0 & 0 & 1 \end{pmatrix}$$. We show that conditions (I), (II), (III), and (IVb) of Theorem 14 are satisfied.

(I) $\ker \Gamma _k$ is generated by the vector $(\frac{k_{32} - 2k_{34}}{k_{21}k_{32} - 2k_{21}k_{34} + k_{23}k_{34}},\frac{k_{23}}{k_{21}k_{32} - 2k_{21}k_{34} + k_{23}k_{34}},\frac{1}{k_{45}})^\textsf{T}$ and hence $\ker \Gamma _k\cap \mathbb {R}^4_{>0}\ne \emptyset$ iff $k_{32} - 2k_{34} >0$.

(II) Satisfied trivially.

(III) Clearly, $d = 3-1-2 = 0$. Since $\dim (K) = \dim ({{\,\textrm{im}\,}}{\Gamma _k})=2$, we get $K=L$.

(IVb) Since $L = \mathbb {R}^2$ holds for the monomial difference subspace (and $L \subseteq S$), we get $L=S$.

Using Theorem 14, we find that there exists a unique positive equilibrium in every stoichiometric compatibility class iff $k_{32} - 2k_{34} >0$.

## Data Availability

No datasets were generated or analyzed during the current study.

## A Proof of Theorem 6

### Proof

In the proof of Theorem 5, we have shown that $\bar{b}_1\cdot \bar{b}_\omega <0$ implies $|Y_c| \ge 1$ for all *c*. We now show the other direction, $|Y_c| \ge 1$ for all *c* implies $\bar{b}_1\cdot \bar{b}_\omega <0$.

Recall that determining $Y_c$ is equivalent to solving $f(t) = c^*$ for $t \in (-1,1)$, where

$$ f(t) = \prod _{i=1}^{\omega } (1+t\bar{q}_i)^{\bar{b}_i} $$

and $1 = \bar{q}_1> \cdots > \bar{q}_\omega =-1$. More explicitly, $f(t) = (1+t)^{\bar{b}_1} \cdot \, \cdots \, \cdot (1-t)^{\bar{b}_\omega } > 0$.

Hence, $|Y_c|\ge 1$ for all *c* is equivalent to $|\{ t \in (-1,1) \mid f(t)=c^* \}| \ge 1$ for all $c^*>0$. Now, for all $\delta >0$, the function *f* attains positive minimum and maximum values on the compact interval $[-1+\delta , 1 - \delta ]$, and *f* being surjective onto $(0,\infty )$ requires $\displaystyle \lim _{t \rightarrow -1} f(t) = 0$ and $\displaystyle \lim _{t \rightarrow 1} f(t) = \infty$ (or vice versa). This is equivalent to $\bar{b}_1 > 0$ and $\bar{b}_\omega < 0$ (or vice versa), that is $\bar{b}_1\cdot \bar{b}_\omega <0$. $\square$

## B Reaction networks

### B.1 Index notation

We explicitly state the incidence and source matrices as well as the (rectangular) Laplacian matrix of a labeled simple digraph (*V*, *E*, *k*), using index notation.

(i) The incidence matrix $I_E \in \mathbb {R}_>^{V \times E}$ is given by
  $$ (I_E)_{i,j \rightarrow j'} = {\left\{ \begin{array}{ll} -1, & \text {if } i = j, \\ 1, & \text {if } i = j', \\ 0, & \text {otherwise.} \end{array}\right. } $$
(ii) The source matrix $I_{E,s} \in \mathbb {R}_>^{V_s \times E}$ is given by
  $$ (I_{E,s})_{i,j \rightarrow j'} = {\left\{ \begin{array}{ll} 1, & \text {if } i = j, \\ 0, & \text {otherwise.} \end{array}\right. } $$
(iii) The rectangular Laplacian matrix $R_k \in \mathbb {R}^{V \times V_s}$ is given by
  $$ (R_k)_{i,j} = {\left\{ \begin{array}{ll} k_{j \rightarrow i}, & \text {if } (j \rightarrow i) \in E, \\ -\sum _{(i \rightarrow i') \in E} k_{i \rightarrow i'}, & \text {if } i = j, \\ 0, & \text {otherwise.} \end{array}\right. } $$
  It can be decomposed as $R_k = I_E {{\,\textrm{diag}\,}}(k) (I_{E,s})^\textsf{T}$.

### B.2 Proof of the second salt theorem

We prove the second salt theorem for rectangular (rather than square) Laplacian matrices.

#### Proof of Lemma 20

Let $T' = \{ 1,\ldots , t' \} \subseteq T$. The equations $R_k \, \hat{q} = 0$ and $R_k \, 1_{V_s} = \beta$ imply

$$\begin{aligned} \sum _{\begin{array}{c} (i \rightarrow i') \in \\ V \setminus T'\rightarrow T' \end{array}} k_{i\rightarrow i'} \, \hat{q}_i - \sum _{\begin{array}{c} (i' \rightarrow i) \in \\ T'\rightarrow V \setminus T' \end{array}} k_{i'\rightarrow i} \, \hat{q}_{i'}&= 0 , \\ \sum _{\begin{array}{c} (i \rightarrow i') \in \\ V \setminus T'\rightarrow T' \end{array}} k_{i\rightarrow i'} - \sum _{\begin{array}{c} (i' \rightarrow i) \in \\ T'\rightarrow V \setminus T' \end{array}} k_{i'\rightarrow i}&= \sum _{i\in T'} \beta _i . \end{aligned}$$

If $T' \subset T$, then the first equation and the order on $\hat{q}$ imply

$$ \hat{q}_{t'+1} \sum _{\begin{array}{c} (i \rightarrow i') \in \\ V {\setminus } T'\rightarrow T' \end{array}} k_{i\rightarrow i'} \ge \sum _{\begin{array}{c} (i \rightarrow i') \in \\ V {\setminus } T'\rightarrow T' \end{array}} k_{i\rightarrow i'} \, \hat{q}_i = \sum _{\begin{array}{c} (i' \rightarrow i) \in \\ T'\rightarrow V {\setminus } T' \end{array}} k_{i'\rightarrow i} \, \hat{q}_{i'} \ge \hat{q}_{t'} \sum _{\begin{array}{c} (i' \rightarrow i) \in \\ T'\rightarrow V {\setminus } T' \end{array}} k_{i'\rightarrow i}, $$

and the second equation and $\hat{q}_{t'} \ge \hat{q}_{t'+1}$ further imply

$$ \sum _{i\in T'} \beta _i = \sum _{\begin{array}{c} (i \rightarrow i') \in \\ V {\setminus } T'\rightarrow T' \end{array}} k_{i\rightarrow i'} - \sum _{\begin{array}{c} (i' \rightarrow i) \in \\ T'\rightarrow V {\setminus } T' \end{array}} k_{i'\rightarrow i} \ge \left( \frac{q_{t'}}{q_{t'+1}} - 1\right) \sum _{\begin{array}{c} (i' \rightarrow i) \in \\ T'\rightarrow V {\setminus } T' \end{array}} k_{i'\rightarrow i} \ge 0. $$

In particular, $\sum _{i\in T'} \beta _i > 0$ if $\hat{q}_{t'} > \hat{q}_{t'+1}$.

If $T' = T$, then $(T \rightarrow V \setminus T) = \emptyset$, since *T* is an absorbing strong component, and

$$ \sum _{i\in T} \beta _i = \sum _{\begin{array}{c} (i \rightarrow i') \in \\ V {\setminus } T\rightarrow T \end{array}} k_{i\rightarrow i'} \ge 0. $$

The sum is zero if and only if $(V \setminus T\rightarrow T) = \emptyset$, that is, $T=V$. $\square$

## Footnotes

1 The reaction network (with rate constants)  defines a mass-action system with associated ODE  Reaction networks are formally introduced in Sect. 4, where we will continue the example.

2 For two vectors $y^1, y^2 \in \mathbb {R}^m_\ge$ with $y^1+ y^2, \, \bar{\lambda } y^1 + (1- \bar{\lambda }) y^2 \in \mathbb {R}^m_>$, consider the maximal minors of the matrices $(y^1 \quad y^2), \, (y^1-y^2 \quad y^1+y^2), \, ({\hat{y}} \quad {\bar{y}}) \in \mathbb {R}^{m \times 2}$. Explicitly, let

$$\begin{aligned} (y^1 \quad y^2) = \begin{pmatrix} a_1 & a_2 \\ b_1 & b_2 \\ \vdots & \vdots \end{pmatrix}, \qquad (y^1-y^2 \quad y^1+y^2) = \begin{pmatrix} a_1-a_2 & a_1+a_2 \\ b_1-b_2 & b_1+b_2 \\ \vdots & \vdots \end{pmatrix}, \\ ({\hat{y}} \quad {\bar{y}}) = \begin{pmatrix} \hat{\lambda }a_1 + (1-\hat{\lambda })a_2 & \bar{\lambda }a_1 + (1-\bar{\lambda })a_2 \\ \hat{\lambda }b_1 + (1-\hat{\lambda })b_2 & \bar{\lambda }b_1 + (1-\bar{\lambda })b_2 \\ \vdots & \vdots \end{pmatrix}. \end{aligned}$$

If $$\hat{\lambda }> \bar{\lambda }$$, then

$$\begin{aligned} {{\,\textrm{sign}\,}}\left( \frac{\hat{\lambda }a_1 + (1-\hat{\lambda })a_2}{\bar{\lambda }a_1 + (1-\bar{\lambda })a_2} - \frac{\hat{\lambda }b_1 + (1-\hat{\lambda })b_2}{\bar{\lambda }b_1 + (1-\bar{\lambda })b_2} \right) = {{\,\textrm{sign}\,}}\left( \frac{a_1-a_2}{a_1+a_2} - \frac{b_1-b_2}{b_1+b_2} \right) = {{\,\textrm{sign}\,}}\begin{vmatrix} a_1&a_2 \\ b_1&b_2\end{vmatrix}. \end{aligned}$$
